# Three-Dimensional Mass Spectrometry Imaging Reveals Distributions of Lipids and the Drug Metabolite Associated with the Enhanced Growth of Colon Cancer Cell Spheroids Treated with Triclosan

**DOI:** 10.1021/acs.analchem.2c00768

**Published:** 2022-09-28

**Authors:** Peisi Xie, Hongna Zhang, Pengfei Wu, Yanyan Chen, Zongwei Cai

**Affiliations:** State Key Laboratory of Environmental and Biological Analysis, Department of Chemistry, Hong Kong Baptist University, Kowloon 999077, Hong Kong SAR, China

## Abstract

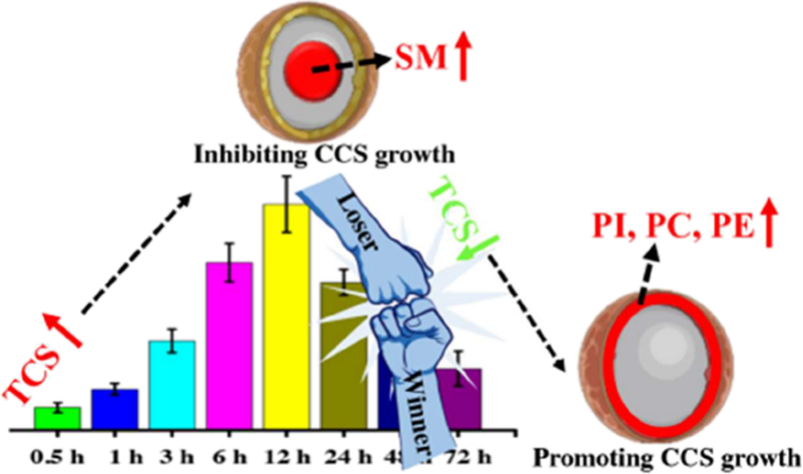

The application of mass spectrometry imaging (MSI) to explore the responses of cancer cell spheroids (CCS) after treatment of exogenous molecules has attracted growing attention. Increasing studies have utilized MSI to image the two-dimensional distributions of exogenous and endogenous molecules in planar CCS sections. However, because CCS are volumetric and heterogenous, maintaining their three-dimensional (3D) information is essential for acquiring a better understanding of the tumor microenvironment and mechanisms of action of exogenous molecules. Here, an established method of 3D MSI was applied to distinguish the distributions of triclosan sulfate and endogenous lipids in three microregions of colon CCS with an enhanced growth induced by the treatment of triclosan, a common antimicrobial agent. The results of 3D MSI showed that triclosan sulfate gradually accumulated from the periphery to the entire structure of CCS and finally localized in the core region. Spatial lipidomics analysis revealed that the upregulated phosphatidylethanolamine (fold change (FD) = 1.26, *p* = 0.0021), phosphatidylinositol (FD = 1.17, *p* = 0.0180), and phosphatidylcholine (FD = 1.22, *p* = 0.0178) species mainly distributed in the outer proliferative region, while the upregulated sphingomyelin (FD = 1.18, *p* = 0.024) species tended to distribute in the inner necrotic region. Our results suggest that a competitive mechanism between inhibiting and promoting CCS growth might be responsible for the proliferation of CCS treated with triclosan.

## Introduction

Three-dimensional (3D) imaging is favorable to be applied in life sciences because biological processes consist of multiple 3D phenomena including the dynamic changes of drug metabolism in targeted organs and the formation and development of highly heterogenous solid tumors.^1^ Current traditional 3D imaging techniques (e.g., positron emission tomography (PET), magnetic resonance imaging (MRI), and ultrasound and single-photon emission computed tomography (SPECT)) have been widely used to display organs or body anatomy by tracing fluid molecules or a particular chemical.^1^ However, these techniques are not useful in spatial lipidomics or proteomics that are composed of techniques of mass spectrometry-based omics and molecular imaging due to their low-throughput character. 3D mass spectrometry imaging (3D MSI) is an innovative, label-free, and in situ molecular imaging technique with a great potential in spatial proteomics and lipidomics.^2^ It allows to identify the localization of hundreds of endogenous (e.g., lipids, peptides, protein) and exogenous (e.g., drugs, environmental pollutants) molecules in one experiment. It has wide coverages and applications in complex biological systems from single cell to whole-body animals.^3,4^ In addition, in common with two-dimensional (2D) MSI, 3D MSI also can be integrated with MS/MS analysis and staining techniques (e.g., immunohistochemistry) for the identification and annotation of biomolecular species.^5^

Traditionally, 2D cancer cell culture has been commonly utilized in anticancer drug screening and environmental toxicological studies.^6^ However, cells that grow in monolayers are unable to mimic the 3D cellular architecture observed in the human body, which may result in inaccurate assessments of the effects of exogenous compounds on tumor tissues. Compared with 2D cancer cell models, 3D cancer cell models such as cancer cell spheroids (CCS) have more similar characteristics including the spatial structure, gene expression levels, cell–cell interaction, growth kinetics, and physical barriers with solid tumors.^7^ For instance, owing to the limited diffusion of nutrients and oxygen, CCS with diameters larger than 500 μm display three cell layers with different cellular states. These three layers contain the outer proliferative area, the middle quiescent area, and the inner necrotic area. Similar internal structures include the parenchyma area containing proliferative and quiescent cells and the necrotic area containing dead cells could also be found in solid tumors. Therefore, in cancer research, CCS have attracted an increasing attention as a promising and valuable in vitro model to evaluate the effect of exogenous molecules on cancer progression.

Since the first study of protein distributions in CCS using matrix-assisted laser desorption/ionization (MALDI) MSI in 2011,^8^ many other studies using MSI have investigated the responses of CCS after treatment of different exogenous molecules.^9−11^ Most of these studies focused on using MSI to image the 2D distributions of exogenous and altered endogenous molecules in planar CCS sections, which lead to the acquirement of the incomplete spatial information of molecules in CCS.^12,13^ One previous study^10^ utilized a total of seven CCS sections to investigate the time-dependent penetration of one anticancer drug in colon CCS. These selected seven sections of one cell spheroid could mimic the 3D structure of the cell spheroid. However, the detailed procedure for establishing the 3D MSI in a stacked way for one cell spheroid or two cell spheroids in drug-treated and -untreated groups is still lacking. The stacked 3D MSI of CCS may contribute to acquiring a better understanding of the tumor microenvironment and mechanisms of action of exogenous molecules.

Triclosan (TCS) is an antimicrobial agent that has been extensively used in over 2000 consumer products, including soaps, mouthwashes, toothpastes, and cosmetics.^14^ Because of the wide use of this agent, it was frequently detected in the human body, raising emerging concern about its impact on human health.^14^ Yang et al. reported that exposure to TCS in mice increased colonic tumorigenesis, tumor size, and colonic inflammation possibly by modulating the toll-like receptor 4 signaling and gut microbiota.^15^ Their in vitro study showed that TCS treatment induced elevated expression levels of proinflammatory chemokines and cytokines in mouse colon cancer cells.^16^ However, little is known about the effects of TCS exposure on colon cancer at the metabolic level.

Lipids are a branch of metabolites that play crucial roles in many cellular functions, such as signal transduction, protein trafficking, and energy storage.^4^ Numerous studies suggested that alterations of lipid metabolism in cancer cells are closely associated with many cellular processes, including cell proliferation, apoptosis, and differentiation.^17^ For example, Zhou et al. utilized graphene oxide as a MALDI matrix to investigate the lipid distribution in mouse breast cancer tissues.^18^ Interestingly, their data revealed that unsaturated fatty acids (FA) with chain lengths of 20–22 carbon atoms (e.g., FA(20:4), FA(22:5), and FA(22:6)) tended to distribute in the necrotic area, while FA with chain lengths of 16–18 carbon atoms (e.g., FA(16:0), FA(18:1), and FA(22:6) tended to distribute in the parenchyma area. One study investigated the effect of an anticancer drug (irinotecan) on the lipid metabolism of HCT116 colon CCS.^17^ It demonstrated that drug treatment induced a significant up-regulation of glycerolipids (GLs, e.g., triglyceride (TG)) and glycerophospholipids (GPs, e.g., phosphatidylcholine (PC), phosphatidylethanolamine (PE), and phosphatidic acid (PA)) that are essential for energy storage and membrane structure. Because of the multifunctionality of lipids and heterogeneity of CCS, a deeper understanding on changes of lipid abundance and distribution in CCS after the treatment of exogenous compounds (e.g., TCS) is essential, which could provide insights into lipid-mediated processes and related toxic mechanisms.

In this present work, CCS generated by HCT116 human colon cancer cells were exposed to TCS to explore its effect on CCS growth. The method of 3D MSI for CCS was established and employed to observe the time-dependent penetration of TCS sulfate (TCSS, one major phase II metabolite of TCS). To explore TCS metabolism in CCS, we performed the quantitative analysis of TCS and TCSS in CCS and culture medium at different time points by using liquid chromatography–tandem mass spectrometry (LC–MS/MS). In addition, 3D MSI combined with MS-based lipidomics was utilized to analyze the variations of distribution and abundance of lipids between control and exposed groups to uncover the effect of enhanced growth of HCT116 CCS treated with TCS.

## Experimental Section

### Chemical and Reagents

Chloroform, ethanol (EtOH), methanol (MeOH), isopropanol (IPA), acetonitrile (ACN), ammonium acetate, ammonium hydroxide solutions (30%), dichloromethane (DCM), and formic acid were purchased from Merck (Darmstadt, Germany). Gibco RPMI Media 1640 (GRM1640), 4-chloro-phenylalanine (4-cl-Phe), fetal bovine serum (FBS), trypsin (0.25%), and penicillin–streptomycin (100 U/mL) were purchased from Thermo Fisher (Cambridge, MA, U.S.A.). *Trans*-2-[3-(4-*tert*-butylphenyl)-2-methyl-2-propenylidene]malononitrile (DCTB), 9-acridinamine (9AA), 1,5-diaminonaphthalene (1,5-DAN), 3-aminoquinoline (3-AQ), and *N*-phenyl-2-naphthylamine (PNA) were purchased from Sigma-Aldrich (St. Louis, MO, U.S.A.). Lysophosphatidylcholine (19:0) (LPC(19:0)) and ceramide (d18:1/17:0) (Cer(d18:1/17:0)) were purchased from Avanti Polar Lipids (Alabaster, AL, U.S.A.). TCS was purchased from TCI Chemicals (Tokyo, Japan). TCS glucuronide (TCSG) and TCS sulfate (TCSS) were synthesized in our laboratory.^19^

### Preparation of CCS and Treatment of TCS

The HCT116 colorectal carcinoma cells (ATCC, Manassas, VA, U.S.A.) were grown in GRM1640 complete medium (Thermo Fisher Scientific, U.S.A.). A total of 200 μL of medium containing 5000 cells was added into each inner well of ultralow attachment 96-well plates (Corning Inc., Cornish, ME, U.S.A.). Plates were placed in an incubator with 5% CO_2_ at 37 °C. After three days in culture, medium (100 μL) was changed every two days.

On day three in culture, CCS were exposed to various concentrations of TCS (final concentrations: 0.1, 0.25, 0.5, 1, 2.5, 5, 10, and 25 μM). A total of 100 μL of culture medium containing different concentrations of TCS was replaced every 48 h. An inverted microscope (Leica, Germany) was used to measure the spheroid area every 48 h at 5× magnification. On day 16 in culture, 0.25% trypsin (100 μL) was added into each well for 60 min. The cell number of each cell spheroid was counted by using an automated cell counter.

### MALDI-MS Analysis

Matrices, PNA (7 mg/mL), and 1,5-DAN (7 mg/mL), were dissolved in pure ACN. DCTB (7 mg/mL) matrices were dissolved in pure DCM. Matrices, 9AA and 3-AQ, were dissolved pure MeOH and EtOH, respectively. To optimize the matrix for detecting TCS, TCSS, and TCSG, a total of 1 μL of different matrices was respectively mixed with 1 μL of standard solutions containing TCS (2 μM), TCSS (2 μM), and TCSG (2 μM). A total of 0.5 μL of the above mixture was added onto a stainless-steel plate. Three replicates were performed for each matrix. The MS spectra were obtained by using a vacuum rapiflex MALDI-TOF Tissuetyper (Bruker Daltonics, Germany) with a laser power (80%) and a scan range (*m/z* 100–1000).

### Establishment of 3D MSI for CCS

Gelatin solution (175 mg/mL in ultrapure water) in a 50 mL tube was placed in a water bath for 30 min at 70 °C. A total of 120 μL of warm gelation solution was added in each well of the 96-well plate by a 200 μL manicured tip (Figure S1A). One cell spheroid was washed with normal saline 3 times and transferred into the well by a new 200 μL manicured tip (Figure S1B). The cell spheroid was moved carefully to the center area of the well by a 10 μL tip. Until the gelatin hardened into a solid, 60 μL of warm gelation was added to cover the cell spheroid (Figure S1C). The plate was stored at −20 °C for 1 h before taking it out for 20 min at room temperature. A 15 cm steel needle was used to scoop the gelatin block out. The block was sliced into 14 μm thick sections. All sections containing cell spheroids were thaw-mounted on an indium tin oxide slide (Figure S1D), dried in a vacuum desiccator, and stored at −80 °C before matrix deposition.

Matrices, DCTB (5 mg/mL) and DHB (15 mg/mL) were dissolved in pure DCM and 90% MeOH, respectively. Matrices were deposited on indium tin oxide (ITO) slides by using an airbrush (Figure S1E). The total of 10 mL of DCTB matrix and 5 mL of DHB matrix were deposited on each ITO slide. The MSI experiment was performed by using a rapifleX MALDI-TOF Tissuetyper equipped with a smartbeam 3D laser (Figure S1F). The laser power, scan range, and resolution were set at 86%, *m/z* 100–1000, and 50 μm, respectively.

For the creation of 3D image of one cell spheroid (Video S1), all raw data of consecutive sections from one spheroid on culture day 15 were imported into SCiLS Lab MVS version 2020b (Bruker Daltonics, Germany). For CCS with diameters around 800–1000 μm, 40–60 sections could be obtained for each spheroid. These sections that belong to one cell spheroid were placed on the same ITO slide. The data were processed by the segmentation analysis under the normalization method of the total ion count. All ion images were processed with the weak-denoising method. According to the obtained segmentation map, we created and renamed the new polygonal region for each CCS section (Figure S1G). The new regions were selected from the section 1 to the last section and overlapped in order without adjusting the *x* and *y* coordinates (Figures S1H and S2A). The regions from section 1 and the middle section were overlapped in order to create the half spheroid (Figure S2B). For the establishment of the quarter spheroid, the clapping plane is moved from the top of the half spheroid (Figure S2C) to the side of the half spheroid (Figure S2D). The clapping plane is moved forward to the middle region of the half spheroid (Figure S2E). The quarter spheroid was shown from the horizon view (Figure S2F). The thickness of all sections was set as 14 μm. The value of the *z* coordinate of “section 1” was set as 14 μm. These values would increase automatically based on the number of overlapped sections.

For the comparison of the differences in the spatial distribution of endogenous lipids between control and TCS-treated groups, the raw data of consecutive sections from half spheroids on culture day 15 in two groups were imported into the software. The new regions were selected from the section 1 of the cell spheroid in the TCS-treated group to the middle section of the cell spheroid in the control group. The regions of the cell spheroid in the TCS-treated group were overlapped in order without adjusting the *x* and *y* coordinates (Figure S1I). When aligning the middle section of the cell spheroid in the TCS-treated group and the section 1 of the cell spheroid in the control group, we adjusted the *y* coordinate to move the section 1 to the right side of the middle section and overlaid the remaining sections in order without any adjusting of two coordinates (Figure S1I and Video S2). In order to obtain more inner distribution information of molecules in cell spheroids, we established the quarter spheroids by moving the clipping plane from outside to inside to cut the half spheroids (Figure S1J and Video S3). The 3D ion images were viewed in the volume mode. The scale bar was set according to the value of *z* coordinate of the cell spheroid.

### Processing and Analysis of MSI Data

MSI data were imported into software of SCiLS Lab 2016a (Bruker Daltonics, Germany) and processed by the unsupervised segmentation analysis. The method of the total ion count was chosen to normalize the data. The minimal interval width was set at ±0.10 Da. All ion images were processed with the weak-denoising method. The probabilistic latent semantic analysis (pLSA) with random initialization was performed to discriminate the metabolic differences among different regions of TCS-treated and control groups. To confirm the significant changed lipids from MALDI-MSI data, statistical comparison of 15 CCS sections from three cell spheroids (five middle sections in one cell spheroid) in each group was performed by using the paired t test. Lipids were identified by MALDI-MS/MS using a timsTOF flex MALDI-2 (Bruker Daltonics, Germany).

## Results and Discussion

### Establishment of 3D MSI for CCS

**Figure 1:**
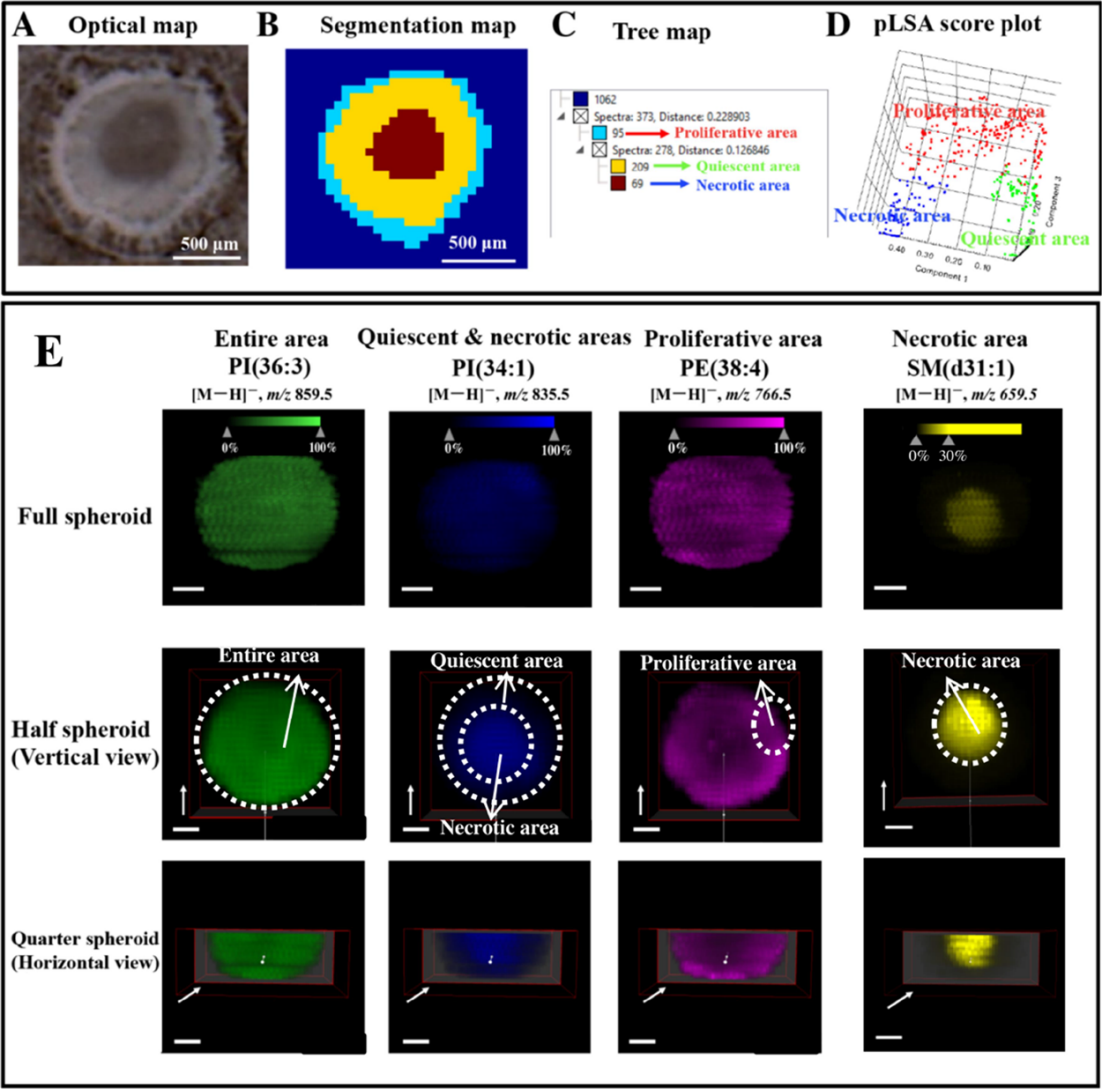
(A) Optical map of one section of the cell spheroid. (B) Segmentation map of one section of the cell spheroid. (C) Tree map. (D) pLSA score plot. (E) Region-specific distribution of lipids in the cell spheroid. The scale bars in all 3D ion images were 200 μm. The direction of the white arrows indicated the moving direction of the clipping planes.

Previous work reported that the cell spheroid contains three areas because of the existence of gradients of oxygen and nutrients.^7^ The existence of the three areas, including the proliferative area (baby blue), quiescent area (yellow), and necrotic area (brown), was proven by the segmentation analysis of one section of the HCT116 cell spheroid (Figure 1A–C). The plots of probabilistic latent semantic analysis (pLSA) at 95% confidence intervals showed that there were clear separations among three areas (Figure 1D), indicating significant differences in metabolic characteristics among cells within three areas. The corresponding loading plot (Figure S3) for the principle component 1 and 2 showed seven ions at *m/z* 8885.5, 886.6, 887.6, 861.5, 835.5, 864.6, and 863.6 that distributed outside of the 95% confidence ellipse. These ions were identified as important variables that contributed to discriminating different areas of CCS. After constructing the 3D model for full cell spheroids, we found that some lipids, such as sphingomyelin(d31:1) (SM(d31:1)), distributed in the necrotic area (Figure 1E, top row). However, in the 3D model for the full cell spheroid, the information of internal distribution of molecules in cell spheroids could not be seen (Figures 1E and S4, top row). Hence, we established the 3D model for the half spheroid (Figure S2B) by overlapping half the number of sections of the cell spheroid. From the vertical view of the half spheroid, we found that some lipids (e.g., phosphatidylinositol (36:3) PI(36:3)) distributed in the entire area and some lipids, such as phosphatidylethanolamine (38:4) (PE(38:4)), PI(40:5), and PI(38:4), predominantly located in the proliferative area (Figures 1E and S4, middle row). In addition, some lipids (e.g., PI(34:1), (PE(O-36:2), PE(O-36:3)) were found to locate in the quiescent and necrotic areas from the view of the full spheroid (Figures 1E and S4, top row). However, from the vertical view of the half spheroid, these lipids were found to distribute more in the necrotic area (Figures 1E and S4, middle row).

The vertical view of the half spheroid provides an accurate information of the horizontal distribution of biomolecules (Figure S2C ,D), but it cannot provide the information of the vertical distribution. Thus, we cut the half spheroid by moving the clapping plane to establish the quarter spheroid (Figure S2E). The results demonstrated that under the horizontal view of the quarter spheroid (Figures 1E and S4, bottom row), some lipids (e.g., PE(38:4), PI(40:5), PI(38:4)) exhibited 3D hollow inner distributions and some lipids (e.g., PI(34:1), (PE(O-36:2), PE(O-36:3)) showed 3D high-intensity central distributions (Figure S5). Taken together, the vertical view of half spheroids and the horizontal view of quarter spheroids were chosen to investigate the distribution of molecules in the following MSI experiments.

### Time-Dependent Distribution of TCSS in Colonic CCS

**Figure 2:**
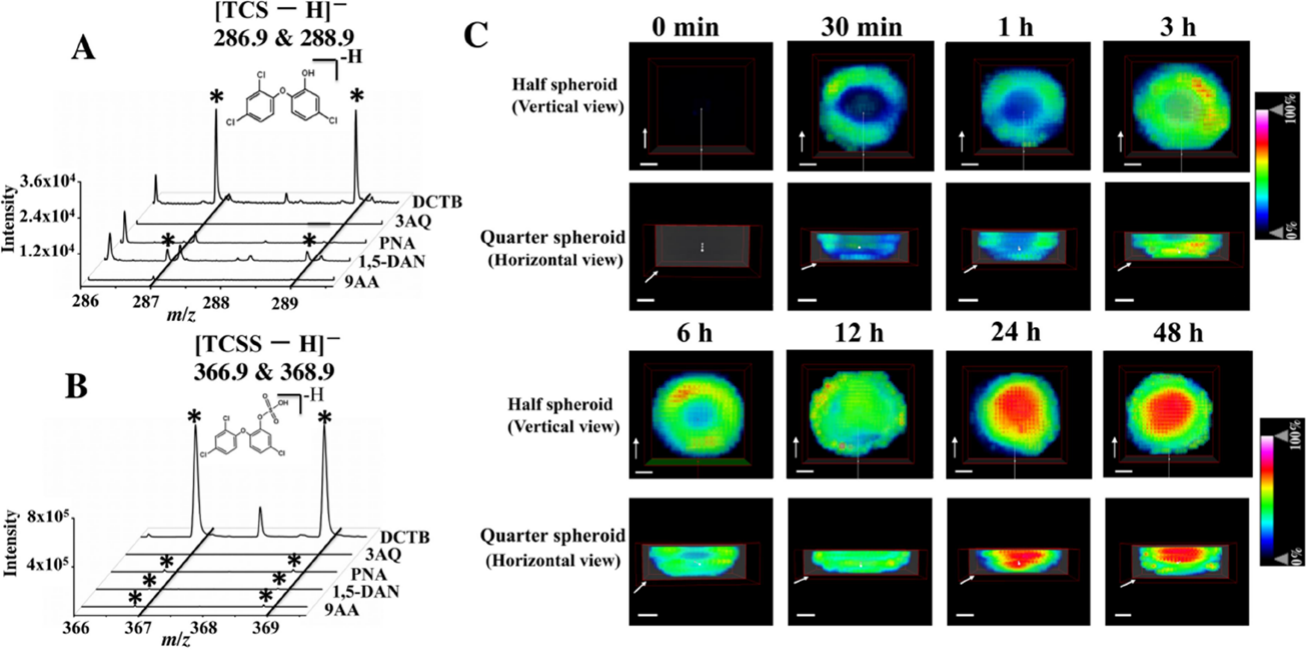
Optimization of matrix and time-dependent distribution of TCSS in colonic CCS. (A) Ion peaks of 1 μM TCS with different matrices. Asterisks indicated detected ion peaks of TCS. (B) Ion peaks of 1 μM TCSS with different matrices. Asterisks indicated detected ion peaks of TCSS. (C) Distribution of the TCSS ion (*m/z* 366.9) in CCS at different time points. Because of the presence of chlorine-35 and chlorine-37, both of [TCS–H]^−^ and [TCSS – H]^−^ have two major ion peaks. The scale bars in all 3D ion images were 200 μm. The direction of the white arrows indicated the moving direction of the clipping planes.

In MALDI analysis, the choice of matrix plays important roles in detecting particular molecules with high and sensitive MS signal responses.^20^ Hence, we optimized matrix to detect 1 μM of TCS and its two major metabolites (TCSS and TCS glucuronide (TCSG)) by using MALDI-TOF MS in negative ionization mode. In this study, 9-aminoacidine (9AA), 1,5- diaminonaphthalene (1,5-DAN), 3-aminoquinoline (3-AQ), *N*-phenyl-2-naphthylamine (PNA), and Trans-2-[3-(4-*tert*-butylphenyl)-2-methyl-2-propenylidene]malononitrile (DCTB) were used. The spectra of these five matrices (Figure S6) showed that no matrix peaks interfered with TCS and TCSS detection. It should be noted that because of the presence of chlorine-35 and chlorine-37, both of [TCS–H]^−^ and [TCSS – H]^−^ have two major ion peaks (Figure 2A, B). TCS-related ions ([TCS–H]^−^ at *m/z* 286.9 and 288.9) were detected in DCTB and 1,5-DAN matrices (Figure 2A). The TCSS-related ions ([TCSS – H]^−^ at *m/z* 366.9 and 368.9) were detected in DCTB, PNA, 1,5-DAN, and 9AA matrices (Figure 2B). The TCSG-related ions were not determined in these five matrices. Among these matrices, DCTB showed the highest intensity signal of deprotonated ions of TCS and TCSS. The intensity signal of [TCSS – H]^−^ ions was about 20-fold higher than that of [TCSS – H]^−^ ions by using the DCTB matrix (Figure 2A, B). In order to confirm the detection ability of DCTB for TCS and TCSS in HCT116 CCS, we acquired the MS spectra on DCTB-sprayed sections of CCS after TCS (10 μM) treatment by using MALDI-TOF MS. However, only the TCSS-related ion could be observed on CCS sections (Figure S7), which may be because TCSS demonstrates a higher signal response compared to TCS. Other possible reasons include that TCS has been largely metabolized to its metabolites (e.g., TCSS). Therefore, the DCTB matrix was chosen to detect TCSS in the following MSI experiments.

The penetration of drugs and their metabolites into tumor tissues is essential for evaluating the effects of drugs on tumor growth.^21^ In order to investigate the distribution of these compounds in tumor tissues, traditional imaging methods such as PET, MRI, and fluorescence imaging (FI) have been used, which either have a low spatial resolution (millimeter level, for example, PET and MRT) or need a time-consuming labeling procedure (e.g., FI).^22^ MALDI-MSI is a label-free imaging technology that provides a better spatial resolution for cancer research.^23^ Therefore, MALDI MSI was employed to map the distribution of TCSS in HCT116 CCS exposed to TCS (10 μM) at different time points. Each time point contained three cell spheroids. Figures 2C, S7, and S8 showed that no signals of [TCSS – H]^−^ were detected in the cell spheroid in the blank group, suggesting that no endogenous metabolites interfered with TCSS detection. At the time points of 30 min, 1 h, 3 h, and 6 h of TCS treatment, TCSS gradually spread into cell spheroids from the periphery region to the inner region. At 12 h of TCS treatment, TCSS distributed in the entire structure of the cell spheroid. At the time points of 24 and 48 h of treatment, TCSS was found to be intense in the core of cell spheroids.

The previous study demonstrated that with increasing exposure time, irinotecan (one anticancer drug) gradually penetrated into HCT116 cell spheroids from the outer region to the center region and eventually distributed in the entire structure of cell spheroids.^10^ Thus, we speculated that TCS might have this similar penetration behavior. The penetration of TCS into CCS is accompanied by the release of TCSS. Owing to the different metabolic rates of three areas in cell spheroids (necrotic area < quiescent area < proliferative area),^24^ TCSS penetrating into the center area might gradually accumulate, while a portion of TCSS in the outer area might be released into the culture medium. This might account for the accumulation of TCSS in the core of cell spheroids after 24 h of TCS treatment.

### TCS Metabolism in Colonic CCS

**Figure 3:**
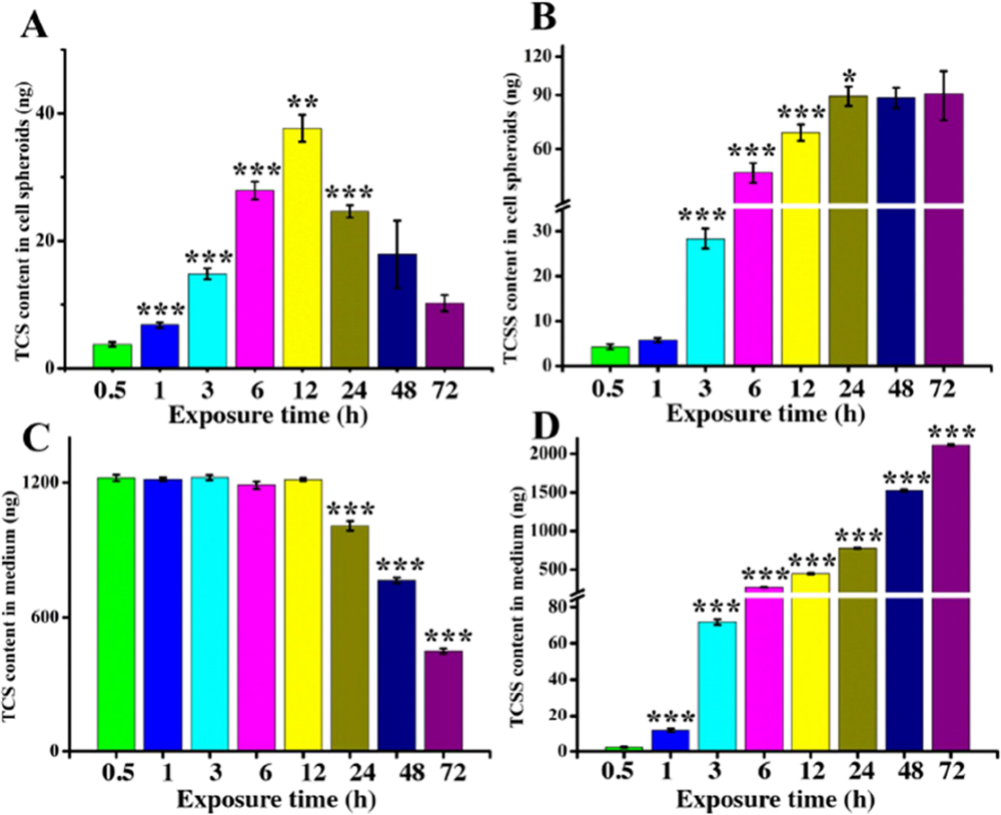
Determination of TCS and TCSS in HCT116 cell spheroids and culture medium at different exposure time. (A) Content of TCS in cell spheroids. (B) Content of TCSS in cell spheroids. (C) Content of TCS in the culture medium. (D) Content of TCSS in the culture medium. Each group contained five sample replicates. Each sample contained five cell spheroids or 1 mL of culture medium. Error bars represent SEM. The statistical analysis was performed between adjacent time points. *p < 0.05, **p < 0.01, ***p < 0.001.

In human living cells, it is generally believed that TCS could be converted into TCSS and TCSG by the phase II metabolism.^19^ In order to confirm this, we first measured the content of TCS, TCSS, and TCSG in culture medium without cell spheroids or with dead cell spheroids. The results (Figure S9) showed that only TCS was detected in culture medium and the content of TCS remained unchanged with increasing time. However, when TCS was added into the culture medium containing living cell spheroids, both TCS and TCSS were detected in culture medium (Figure 3), suggesting that TCSS could be produced by TCS only in the presence of living cell spheroids. The content of TCSG in culture medium was below the instrumental detection limit.

To investigate the metabolism of TCS in HCT116 CCS, we further used LC–MS/MS to measure the content of TCS and TCSS in CCS and culture medium at different exposure time. The MS spectra of TCS and TCSS in blank samples of CCS and culture medium (Figure S10) showed negligible signals of TCS and TCSS ions, suggesting that no endogenous compounds interfered with TCS and TCSS detection. As shown in Figure 3B and Table S1, the content of TCSS in CCS gradually increased and then reached a plateau after 24 h of TCS treatment. The content of TCSS in the culture medium augmented gradually (Figure 3D and Table S1), which may be due to the continuous release process of TCSS from CCS. For TCS in CCS, its content gradually increased and then gradually reduced after 12 h of treatment (Figure 3A and Table S1), while the content of TCS in the medium remained unchanged and then decreased after 12 h of treatment (Figure 3C and Table S1). This may be because before 12 h of treatment, the metabolic rate of TCS is slower than its penetrating rate. Meanwhile, as the content of TCS in culture medium is far more than that in CCS, the biotransformation of TCS in CCS would not lead to an obvious decreasing content of TCS in the medium. However, after 12 h of treatment, the penetrating rate of TCS may be slower than its metabolic rate, which leads to the reduced content of TCS in both CCS and culture medium.

### CCS Growth after TCS Exposure

**Figure 4:**
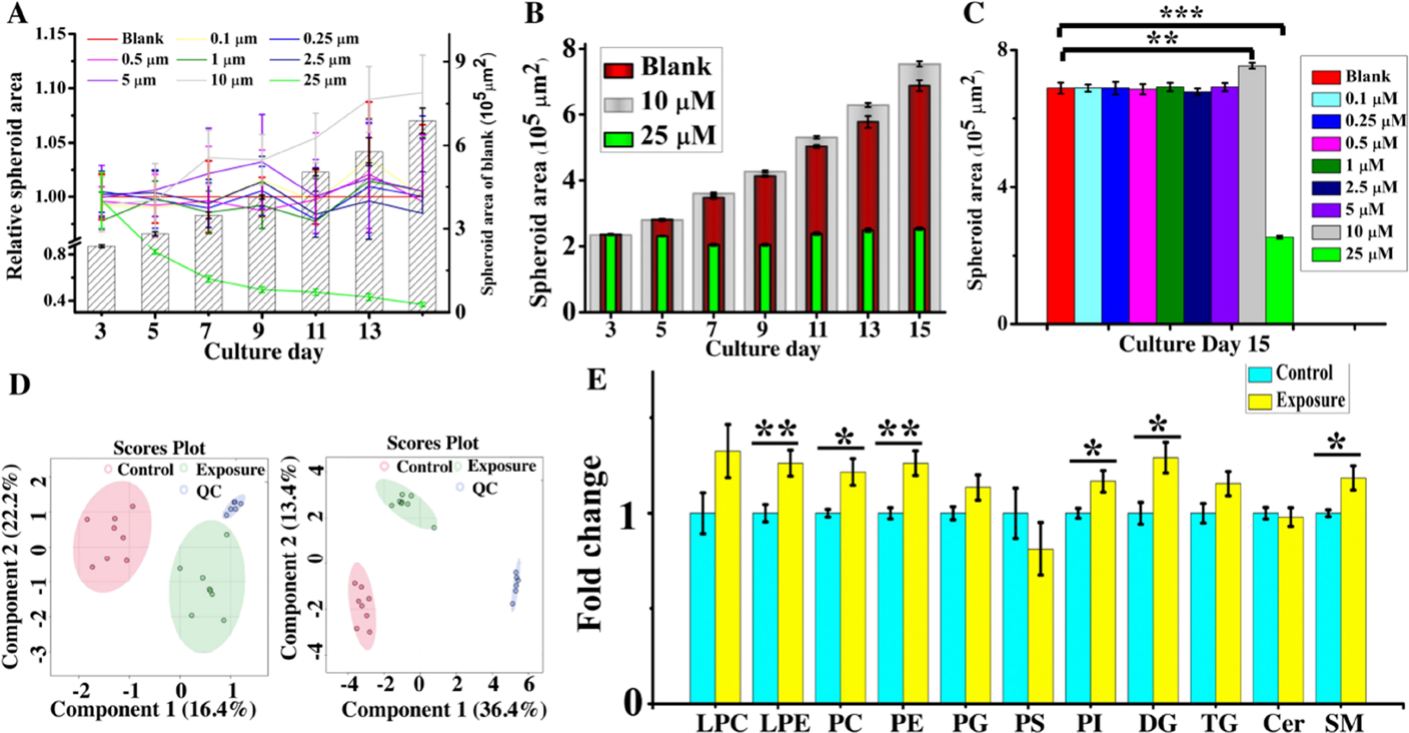
Effects of TCS exposure on CCS growth and lipidomic analysis of HCT116 CCS treated with TCS. (A) Growth curves of HCT116 CCS exposed to various concentrations of TCS (*n* = 8). Spheroid areas of exposure groups relative to the blank group were delineated with line plots. (B) Areas of spheroids treated with 0, 10, and 25 μM TCS at different culture day. (C) Areas of HCT116 CCS exposed to various concentrations of TCS on day 15 in culture (*n* = 8). (D) PLSDA analysis in negative and positive ionization modes (*n* = 8). (E) Fold changes of different lipid classes (*n* = 8). TCS (10 and 0 μM )-treated CCS on day 15 in culture were used for lipidomic analysis. The error bars represented SEM. (*p < 0.05, **p < 0.01, ***p < 0.001).

In order to assess the effect of TCS on the growth of HCT116 CCS, CCS were treated with different concentrations of TCS from day 3 to day 15 in culture. As shown in Figure 4A, the area of HCT116 CCS in the control group gradually increased. The relative area in the 25 μM TCS-treated group gradually decreased, while a slow increase in the relative area was found in the 10 μM TCS-treated group (Figure 4B). On day 15 in culture, we found that the spheroid area in the 10 μM TCS-treated group was significantly larger than that in the blank group (Figure 4C). However, a smaller spheroid area was observed in the 25 μM TCS-treated group than in the blank group (Figure 4C). We further counted the cell number in different groups. The results showed that the cell number in the 10 μM TCS-treated group was significantly more than that in the blank group, while a lesser number of cells was found in the 25 μM TCS-treated group than in the blank group (Figure S11). Taken together, all these results indicated that the treatment of HCT116 CCS with 10 μM TCS induced cell proliferation, while treatment of HCT116 CCS with 25 μM TCS caused an inhibition of cell growth. The inhibition effect may be due to the cytotoxic effect of high-dose TCS exposure. Our results are similar to the results reported in one previous study. In this study, the treatment of mice (chemical-induced colon cancer model) with TCS at environmentally relevant concentrations significantly increased the colitis-associated colon tumorigeneis and tumor size.^15^ In addition, TCS could also promote the proliferation of hepatocytes, lead to hepatocyte fibrosis, and act as a liver tumor promoter.^25,26^ It should be noted that the treatment level (10 μM) used in our study is lower than that (13 μM) detected in human urine.^27,28^ Hence, 10 μM was chosen as the environmentally relevant exposure concentration for further lipidomic and MSI analysis.

### Lipidomic Analysis of HCT116 CCS after TCS Exposure

Lipids, a branch of metabolites, play major roles in biological processes including the constitution of cellular membranes, energy storage, and cell signal transduction.^29^ Previous studies demonstrated that lipid disorders are closely associated with tumor growth and development.^30,31^ Thus, in order to investigate the proliferative mechanisms modulated by lipid metabolism, we performed the lipidomic analysis of HCT116 CCS on day 15 from the control and exposure (10 μM TCS-treated CCS) groups by using LC–MS/MS. The results of the partial least-squares discriminant analysis (PLDSA) showed clear separations between two groups in both negative and positive ionization modes (Figure 4D). A stable instrumental performance was confirmed by the clustered QC samples (Figure 4D). A total of 589 lipids were detected. Among these lipids, a total of 137 lipids (Table S2) were found significantly changed according to the threshold of the fold of change (FC, < 0.8 or > 1.2) and the *p* value (*p* < 0.05). Among these lipids, a total of 55 lipids belonged to glycerophospholipids, consisting of 24 PE, 20 phosphatidylcholines (PC), 4 PI, 4 lysophosphatidylcholines (LPC), 1 phosphatidylglycerol (PG), and 2 lysophosphatidylethanolamines (LPE). A total of 19 lipids were sphingolipids, including 15 sphingomyelins (SM) and 4 ceramides (Cer). The rest lipids were glycerolipids, involving 39 triglycerides (TG) and 24 diacylglycerols (DG). The MS/MS information of some of these lipids is listed in Figure S12.

Given that different lipid classes have different biological functions,^29^ we compared the levels of different lipid classes in the control and TCS-treated groups. The results showed that significant elevated levels of PC, PE, SM, PI, LPE, and DG were found after TCS exposure (Figure 4E). Among these lipid classes, PC and PE are two main glycerophospholipids in cell membranes of all mammals. It has been shown that cancer cells exhibit an enhanced synthesis of PC and PE acting as building blocks of plasma membranes in order to achieve fast cell proliferation.^32^ PI lipid species are required for the activation of the phosphatidylinositol 3-kinase (PI3K) signaling pathway that involves in mediating cell proliferation and survival.^33^ SM is also found in the animal cell membrane, which plays a major role in regulating cell inflammation and apoptosis. It can be synthesized by transferring the phosphorylcholine from PC to ceramide (Cer) in the presence of SM synthase.^34^ Numerous studies demonstrated that administration of SM to mice could inhibit the tumorigenesis and growth of colon cancer.^35−37^ Thus, we speculated that TCS exposure induced the proliferation of colonic CCS, which may be through the competition between inhibiting and promoting CCS growth.

### MALDI-MSI Analysis of HCT116 CCS after TCS Exposure

**Figure 5:**
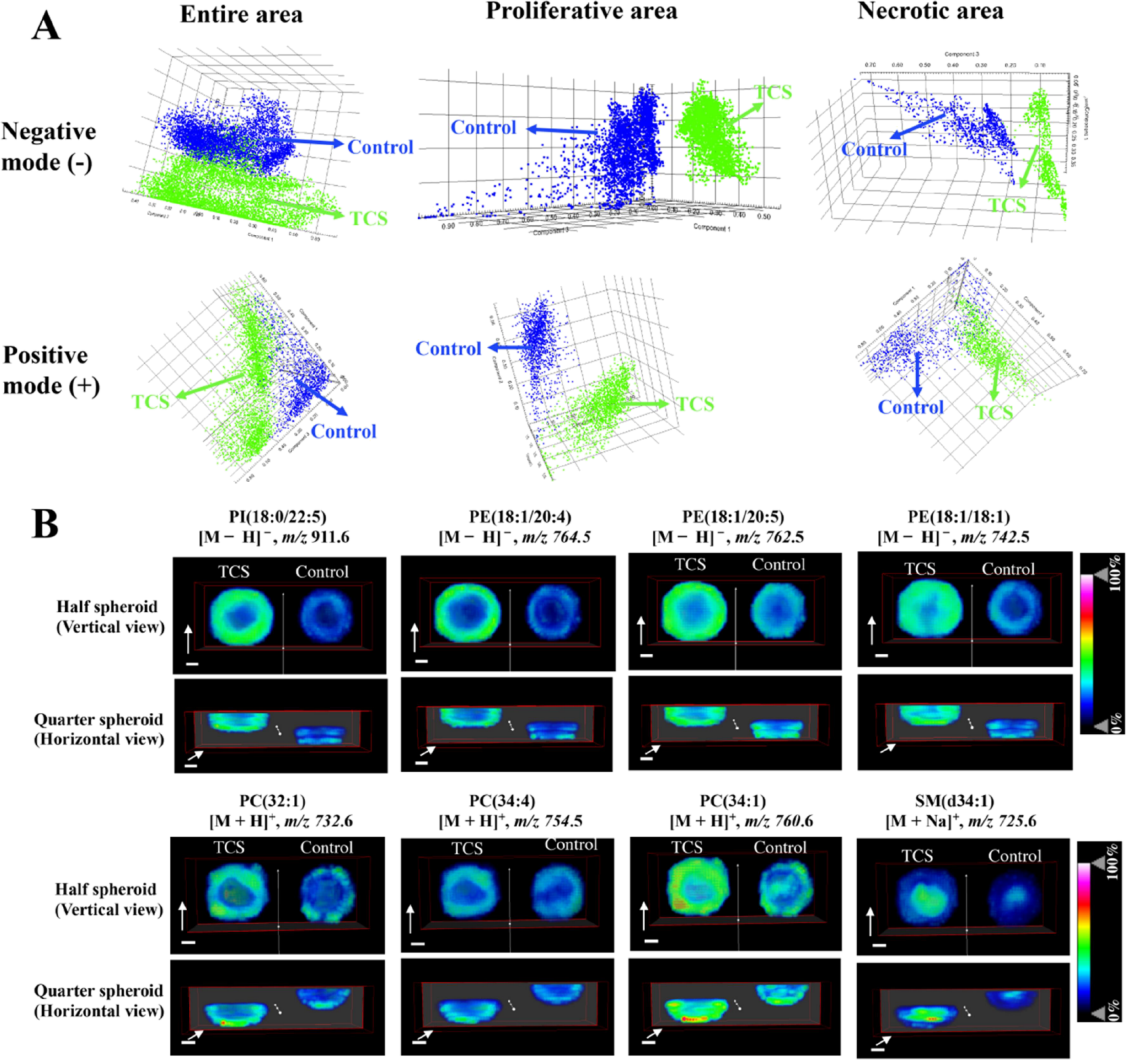
(A) pLSA score plots of MALDI-MSI profiles acquired from the entire area, necrotic area, and proliferative area (*n* = 15). (B) Representative ion images of lipids in the control and TCS-treated groups. Each group contained three biological replicates. The scale bars in all 3D ion images were 200 μm. The range of the direction of the white arrows indicated the moving direction of the clipping planes.

MS-based lipidomics has been widely employed in cancer research aimed at finding new biomarkers and mechanisms of drug action.^38^ In this technique, biological samples were crushed and homogenized to extract lipids, which lead to the loss of information of spatial distribution of lipids in biological samples. This information might play an important role in clarifying the mechanisms of drug action. Hence, MSI was applied to discover a deeper understanding of the proliferative mechanism of HCT116 CCS induced by TCS exposure. We initially further performed the analysis of the lipid profile from three areas (entire area, proliferative area, and necrotic area) of CCS sections to explore the changes in lipid levels between the blank group and the TCS-treated group. The results of probabilistic latent semantic analysis (pLSA) score plots showed that similar to the results of LC data (Figure 4D), there were clear separations in these three areas of CCS sections between the control and TCS (10 μM)-treated groups in both negative and positive ionization modes (Figure 5A), suggesting an obvious alternation in lipid levels after TCS exposure.

LC–MS/MS data (Figure 4E) showed the upregulated levels of six lipid classes (PE, PI, LPE, PC, DG, and SM) in CCS treated with TCS. In order to reveal the changes in the spatial distribution of lipid species in these six classes, the method of 3D MSI for CCS on culture day 15 in control and TCS (10 μM)-treated groups was employed. A total of 17 significantly changed lipid species (Figures 5 and S13) belonging to PE, PI, PC, and SM were identified by MALDI-MS/MS (Figure S14). The statistical analysis of intensities of these 17 lipids between control and TCS-treated groups is shown in Figure S15 and Table S3. In negative ionization mode, the signal intensities of eight PE lipid species in the TCS-treated group were significantly higher than those in the blank group (Figures 5B, S13, and S15). Among these lipids, seven lipids (PE(18:1/20:4), PE(18:1/20:5), PE(18:1/18:2), PE(18:1p/20:4), PE(18:1/18:1), PE(16:0/20:4), and PE(16:0/18:1) mainly distributed in the outer region of CCS, while one lipid (PE(18:0/18:1) distributed in the entire region of CCS. For PI, two upregulated lipids (PI(18:0/22:5) and PI(18:0/20:4)) showed high intensity distributions in the proliferative area of CCS, while one upregulated lipid (PI(18:0/18:1)) showed a high intensity distribution in the inner region of CCS (Figures 5B, S13, and S15). In the positive ionization mode, compared with the blank group, five PC lipid species in the TCS-treated group showed higher signal intensities (Figures 5B, S13, and S15). Among these lipids, four lipids including PC(32:1), PC(34:4), PC(34:1), and PC(36:2) tended to distribute more in the outer region of CCS, while one lipid (PC(36:1)) mainly distributed in the inner region. For SM with an elevated level, it was predominantly found in the inner region of CCS (Figures 5B and S15). This result was similar to that of our previous work, which showed that all detected seven SMs were found to predominantly locate in the necrotic area in liver CCS.^39^ Taken together, these results showed that most of the upregulated lipids (PE, PI, and PC) mainly distributed in the outer proliferative region, while SM with elevated levels tended to distribute in the inner necrotic region.

Taken together, we speculated that the accumulation of TCS in colonic CCS (Figure 3A) would inhibit cell growth by increasing the levels of SM in the inner region of CCS (Figure 5B). However, cells have their ways to protect themselves. They can convert toxic chemicals (TCS) into nontoxic chemicals (TCSS) by the phase II metabolism. Hence, after 12 h of TCS treatment, we can see that cells in CCS tend to consume more TCS (Figure 3A) and store nontoxic TCSS (Figure 2C and Figure 3B). Also, a low concentration of TCS within CCS may promote CCS growth. In the process of CCS growth, an enhanced synthesis of PC and PE in the outer region of CCS (Figure 5B) to build more cell membrane is required. Besides, elevated levels of PI in the outer region of CCS (Figure 5B) may also promote cancer cell proliferation by the activation of the PI3K signaling pathway. Thus, the proliferative mechanism of HCT116 CCS treated with TCS may involve the competition between inhibiting and promoting CCS growth, which is indicated by the fluctuant content of TCS in CCS, the accumulation of TCSS in CCS, upregulated levels of SM in the inner region of CCS, and elevated levels of PC, PE, and PI in the outer region of CCS.

## Conclusions

A method of 3D MSI for CCS was developed and applied to investigate the proliferative mechanism of HCT116 CCS induced by TCS exposure. The time-dependent distribution of TCSS showed that TCSS gradually penetrated from the outer region to the entire region of CCS and finally concentrated in the inner region of CCS. The integrated analysis of MALDI-MSI and LC–MS/MS revealed that TCS exposure caused obvious metabolic disorders and asymmetric metabolite distributions in HCT116 CCS. The 3D MSI results showed that most of the upregulated lipids (PE, PC, and PI) were observed to be intense in the proliferative area of CCS, while SM with elevated levels predominantly distributed in the necrotic area of CCS. These results suggested that a mechanism between inhibiting and promoting colonic CCS growth might be responsible for the proliferation of HCT116 CCS treated with TCS. Thus, the established method of 3D MSI for CCS might offer a comprehensive and accurate evaluation for the effect of exogenous compounds on cancer cells.

## Supporting Information Available

Details of experimental methods (extraction of lipids, instrumental and data analysis; determination of TCS and its phase II metabolites in cell spheroids and culture medium) and additional data (Figures S1–S15, Tables S1–S3 (PDF)
Video of full spheroid (AVI)
Video of half spheroids (AVI)
Video of quarter spheroids (AVI)

## Supplementary Material

ac2c00768_si_001.pdf

ac2c00768_si_002.avi

ac2c00768_si_003.avi

ac2c00768_si_004.avi
