# Supplementary material for: Three-Dimensional Mass Spectrometry Imaging Reveals Distributions of Lipids and the Drug Metabolite Associated with the Enhanced Growth of Colon Cancer Cell Spheroids Treated with Triclosan
Source: Anal Chem. 2022 Sep 28;94(40):13667–75. doi: 10.1021/acs.analchem.2c00768 (PMC9558077; doi:10.1021/acs.analchem.2c00768)
Supplement: Supplementary file 1 — ac2c00768_si_001.pdf [file ac2c00768_si_001.pdf]

Supporting information

Three-dimensional mass spectrometry imaging reveals distributions of lipids and the drug metabolite associated with the enhanced growth of colon cancer cell spheroids treated with triclosan

Peisi Xie[†], Hongna Zhang[†], Pengfei Wu, Yanyan Chen[‡], Zongwei Cai[†*]

[†]State Key Laboratory of Environmental and Biological Analysis, Department of Chemistry, Hong Kong Baptist University, Hong Kong SAR, China

Corresponding author:

Prof. Zongwei Cai, Tel: +852-34117070; Fax: +852-34117348; Email: zwcai@hkbu.edu.hk

Table of Contents

Text S1 Extraction of lipids, instrumental and data analysis..………..S4

Text S6 Determination of TCS and its phase II metabolites in HCT116 CCS and culture medium…………………………………………………………………………….S5

Figure S1 Workflow of the method for establishing 3D MSI for the cell spheroid……........S7

Figure S2 Workflow of constructing 3D MSI from the full spheroid to the half and quarter spheroids...S8

Figure S3 The loading plot for the fist two pricinple compoments.......................................S9

Figure S4 Region-specific distribution of lipids in cell spheroids.......................................S10

Figure S5 Statistical analysis of intensities of three different regions in cell spheroids.......S11

Figure S6 MALDI spectra of five matrices…………………………………………...S12

Figure S7 Representative MALDI-MS spectra of TCSS in CCS treated with TCS at different time points……………………………………………………………………...S13

Figure S8 The distribution of TCSS ion (*m/z* 368.9) in CCS at different time points…......S14

Figure S9 TCS content in culture medium…………………………………….....…S15

Figure S10 MS spectra of TCS and TCSS in samples of standards, CCS blank and medium blank……………………………………………………………………………S16

Figure S11 Cell numbers of HCT116 CCS exposed to various concentrations of TCS on day 16 in culture………………………………………………………………………S17

Figure S12 The chromatogram and MS/MS information of lipid species that were significantly changed……………………………………………………………..S18

Figure S13 Ion images of lipids in the in the control and TCS-treated groups……..............S19

Figure S14 Lipids identified by MALDI-MS/MS using the timsTOF flex MALDI-2 instrument……………………………………………………………………………...S20

Figure S15 Statistical analysis of intensities of different lipids between control and TCS-treated groups...S23

Table S1 Information of statistical analysis of TCS and TCSS in cell spheroids and culture medium at different exposure time..S28

Table S2 Information of lipid markers identified by UPLC-MS/MS. …………………......S30

Table S3 Information of lipid markers identified by MALDI MSI..................................... S37

References……………………………………………………………………………..S38

Text S1 Extraction of lipids , instrumental and data analysis

After confirming the effect of TCS exposure on CCS growth, CCS were cultured in GRM1640 medium containing TCS (10 μM) or 0.1% DMSO from day 3 to day 15 in culture. On day 15, CCS in TCS-treated group and control group were collected and washed three times with PBS. Each group contained eight sample replicates. Each sample included 10-15 cell spheroids. A total of 750 μL of 80% MeOH were added into each sample. The sample was crushed and underwent five freeze-thaw cycles of cell lysis in liquid nitrogen. Chloroform (450 μL) were added followed by mixing and adding ultrapure water (150 μL). The mixture was vortexed (1 min), incubated (5 min) at 25 ˚C and centrifuged (15 min, 12 000 rpm) at −6 ˚C. The liquids in the bottom layer containing lipids and the protein in the middle layer were collected into different tubes and dried in a freezer drier. The lipid residue was dissolved in 100 μL of ACN/IPA/water (65:30:5, v/v/v) containing 2 μg/mL of LPC (19:0) and Cer(d18:1/17:0). The solution was sonicated, vortexed and centrifuged (5 min, 8 000 rpm) at 4 ˚C. The supernatants (80 μL) were used for further lipid analysis. The protein content of each sample was measured by using BCA protein assay.

The lipids were analyzed by aUPLC system coupled with an Orbitrap Fusion Tribrid Mass Spectrometer (Thermo Fisher Scientific, U.S.A.). The details of the analytical methods were described in our previous work.[1,2] Briefly, chromatographic separation was conducted by

using an Hypersil Gold C18 column (2.1 mm × 100 mm; 1.7μm; Thermol Fisher). The column and sampler temperature were set at 50 ℃ and 8 ℃, respectively. The flow rate of mobile phases that contain phase A (ACN/H_2O (60:40, v/v) with 0.1% formic acid and 10 mM ammonium formate) and phase B (ACN/IPA (90:10, v/v) with 0.1% formic acid and 10 mM ammonium formate) was 0.26 mL/min. The injection volume was 10 μL. The gradient was as follows: 0–1 min for 70% B; 1–2 min for 70%–55% B; 2–7 min for 55%–30% B; 7–9 min for 30%–15% B; 9–17 min for 15%–60% B; 17–19 min for 60% B; 19–20 min for 60%–70% B; 20–24 min for 70% B. The main MS parameters included ion transfer tube temperature (285 ℃), spray voltage (3.0 kV), vaporizer temperature (300 ℃), sheath gas (50 units), auxiliary gas (15 units), resolution (120 000 for MS and 30 000 for MS^2) and scan rage (100-1 200). QC samples and blank samples were inserted into the beginning and the end of the running sequence. One QC sample was also inserted into every eight samples.

The LC-MS/MS lipid raw data were imported into Lipidsearch4.0 software (Thermo Fisher Scientific, U.S.A.). The tolerance for precursor and product ions were set as 5.0 ppm. The detected ion adduct forms were $[M – H]^-$, $[M – Cl]^-$ and $[M – HCOO]^-$ for negative ionization mode and $[M + H]^+$, $[M + NH_4]^+$, $[M + Na]^+$ and $[M + K]^+$ for positive ionization mode. The peak areas of all lipids in different lipid classes except diglyceride (DG) and triglyceride (TG) were normalized by the peak area of internal standards and the protein content. More

specifically, LPC(19:0) and Cer(d18:1/17:0) were used to normalize glycerophospholipids (LPC, lysophosphatidylethanolamine (LPE), phosphatidylcholine (PC), phosphatidylinositol (PI), phosphatidylethanolamine (PE), phosphatidylserine (PS) and phosphatidylglycerols (PG)) and sphingolipids (Cer and sphingomyelin (SM)), respectively. Glycerolipids including DG and TG in each sample were normalized by their protein contents. The normalized data were imported into MetaboAnalyst for PLS-DA and heatmap analysis. Significant changed lipids were chosen based on the p value ($p < 0.05$) and the fold change (FC, FC > 0.8 or FC < 1.2). The final data were expressed as mean ± standard deviation (SD) or standard error mean (SEM).

Text S2 Determination of TCS and TCSS in HCT116 CCS and culture medium

For the investigation of TCS metabolism in CCS, CCS on day 15 in culture were exposed to TCS (10 μM) for 0 min, 30 min, 1h, 3h, 6h, 12h, 24h, 48h, and 72h. The group of CCS exposed to TCS (10 μM) for 0 min was set as the control group. The other two control groups included culture medium containing TCS (10 μM) without CCS or with dead CCS. CCS were killed by a 2-h UV irradiation. The 96-well plates containing these two groups were placed in the incubator for 0, 24, 48 and 72h. Each well contained 200 μl of culture medium. A total of five cell spheroids and their culture medium were collected in one 1.5 mL tube and one 2.0 mL tube, respectively. The extraction methods of TCS and its phase II

metabolites in cell spheroids and culture medium were described in our previous works with some modifications.[1,2] Briefly, cell spheroids in one 1.5 mL tube were washed three times with PBS. A total of 450 μL of 80% MeOH and a scope of small steel balls were added into the tube. The mixture was crushed and underwent five freeze–thawing cycles with liquid nitrogen. After centrifuging (10 min, 4 °C, 15 000 g), the supernatants were collected and dried under vacuum. A total of 200 μL of 50% MeOH were added to dissolve the residues. The mixture was centrifuged (10 min, 4 °C, 15 000 g) and the upper solution (150 μL) was used for UPLC-MS/MS analysis. For the extraction of TCS and its metabolites in the culture medium, the culture medium in 2.0 mL tube was centrifuged (5 min, 12 °C, 15 000 g). The supernatants were collected and freeze-dried under vacuum. The residue was dissolved in 500 μL of MeOH and 150 μL of the upper solution were used for LC-MS/MS analysis. The quantitative analysis of TCS and TCSS in the supernatant was performed on a UPLC system coupled to a TSQ Quantiva Triple Quadrupole Mass Spectrometer.

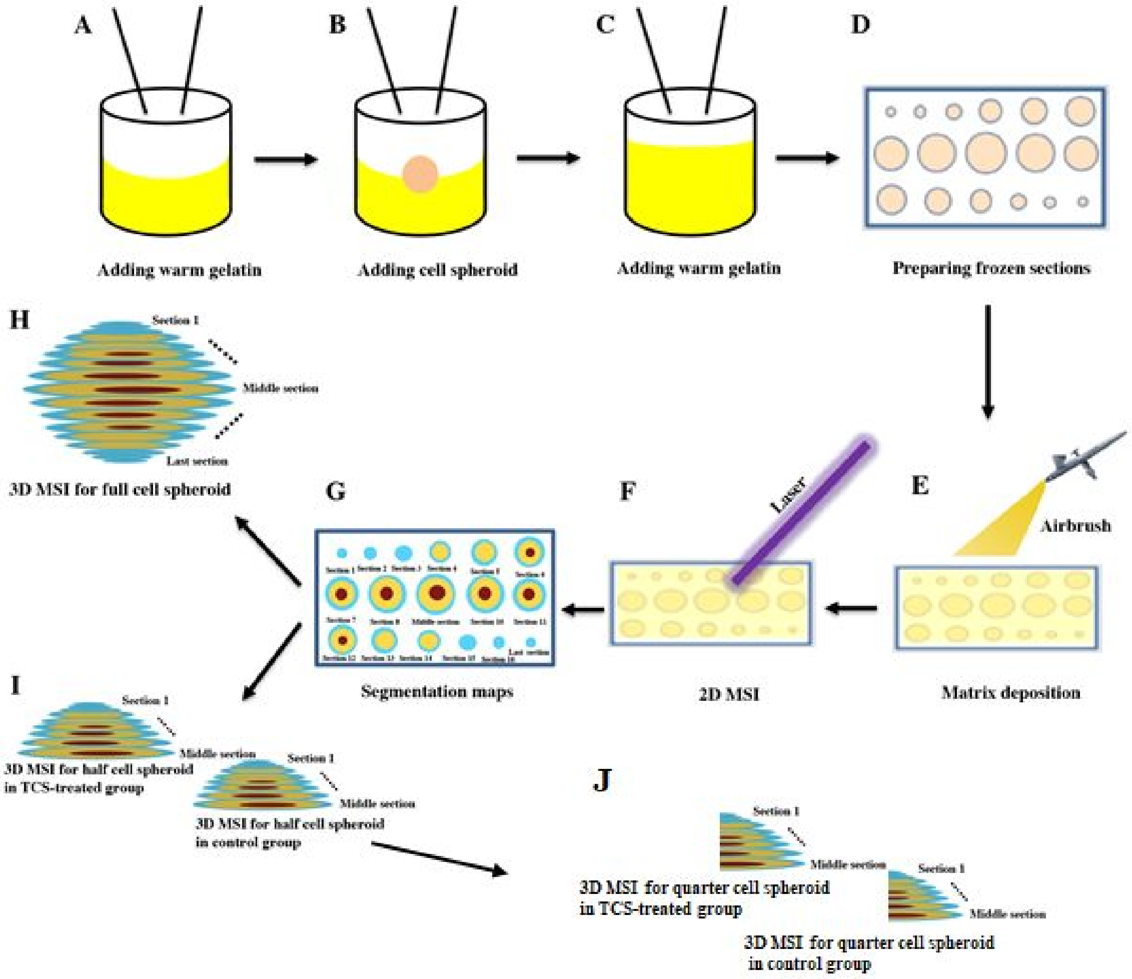

Figure S1. Workflow of the method for establishing 3D MSI for the cell spheroid. (A) Adding warm gelatin. (B) Adding one cell spheroid. (C) Adding warm gelatin to cover the cell spheroid. (D) Preparing frozen sections of the cell spheroid. E) Matrix deposition by using an airbrush. (F) Acquiring 2D MSI data. (G) Segmentation analysis for sections of the cell spheroid. (H) 3D MSI for the full spheroid. (I) 3D MSI for half cell spheroids in the exposure and control groups. (J) 3D MSI for quarter cell spheroids in the exposure and control groups.

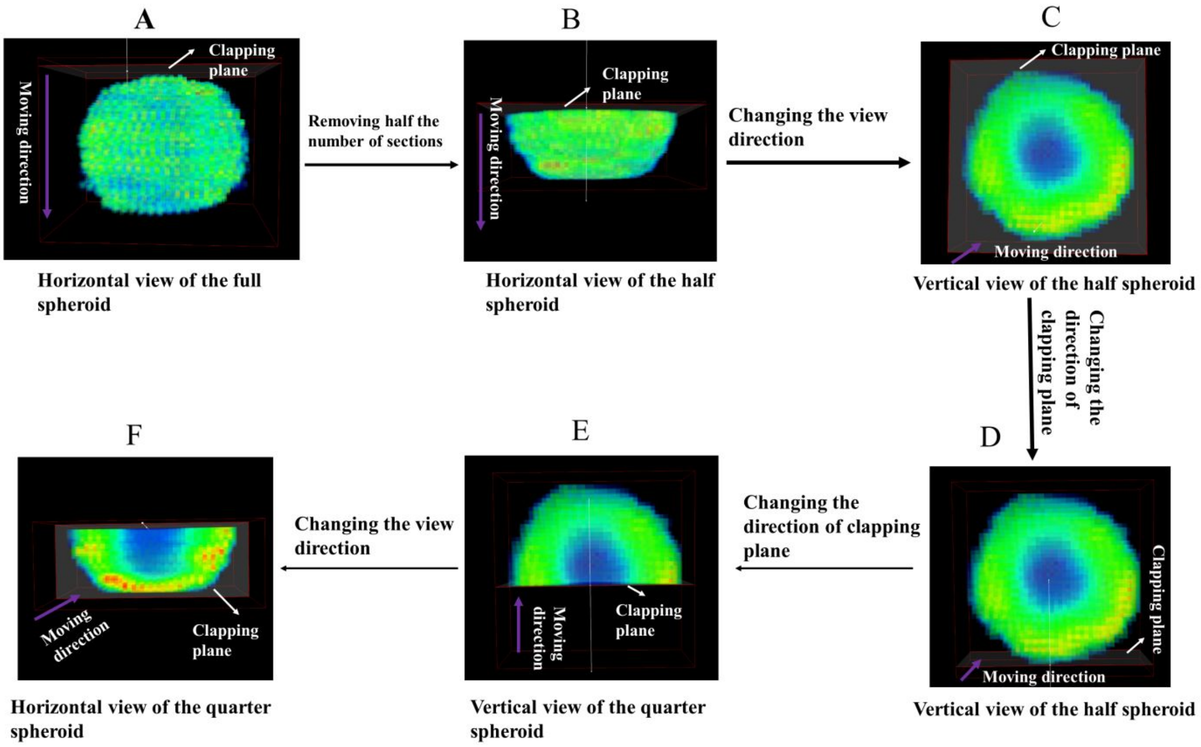

Figure S2. Workflow of constructing 3D MSI from the full spheroid to the half and quarter spheroids. (A) Horizontal view of the full spheroid. (B) Horizontal view of the half spheroid. (C) Vertical view of the half spheroid. (D) Vertical view of the half spheroid. (E) Vertical view of the quarter spheroid. (F) Horizontal view of the quarter spheroid. The full spheroid is made by overlapping all sections of the cell spheroid. The half spheroid is made by overlapping half the number of sections of the cell spheroid. For the establishment of the quarter spheroid, the clapping plane is moved from the top of the half spheroid (C) to the side of the half spheroid (D). The clapping plane is moved forward to the middle region of the half spheroid (E). The quarter spheroid was shown from the horizon view (F). The purple arrow indicates the moving direction of the clapping plane.

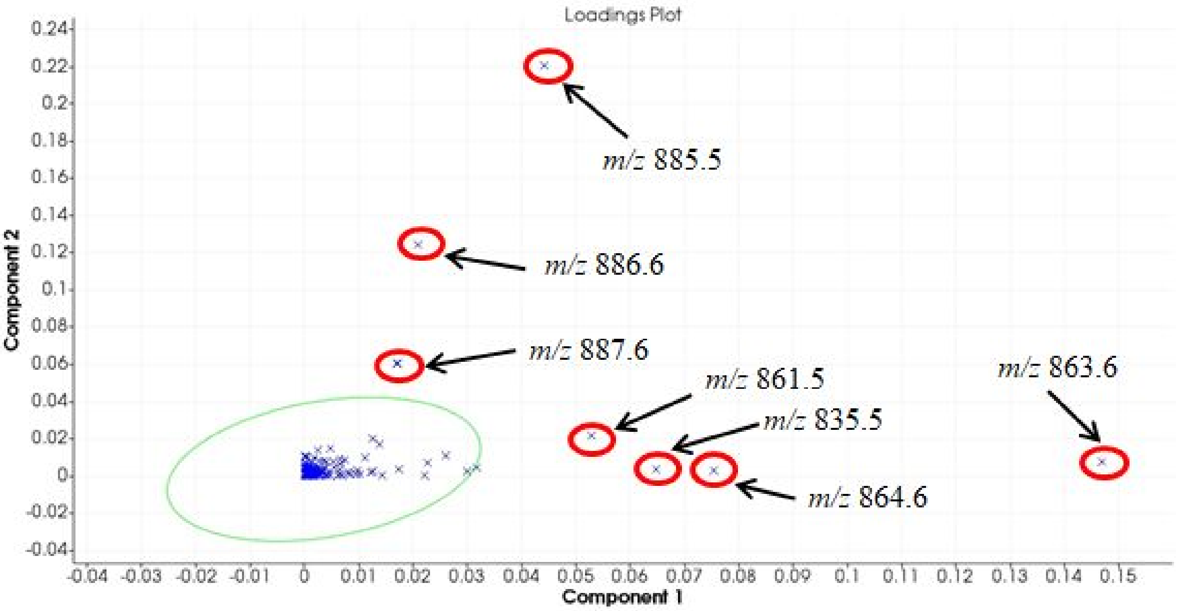

Figure S3. The loading plot for the fist two pricinple compoments (1 and 2). The arrows indicated the ions that did not distribute in the 95% confidence ellipse.

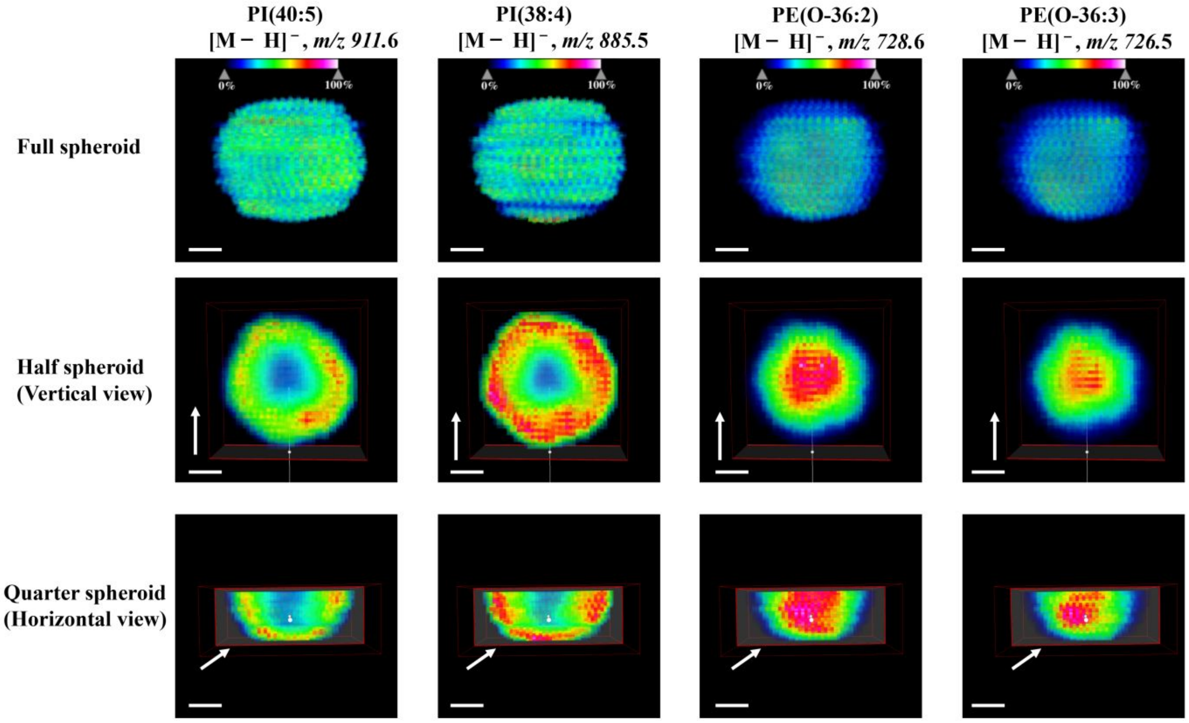

Figure S4. Region-specific distribution of lipids in cell spheroids. The scale bars in all 3D ion images are 200 μm. The direction of the white arrows indicates the moving direction of the clipping planes.

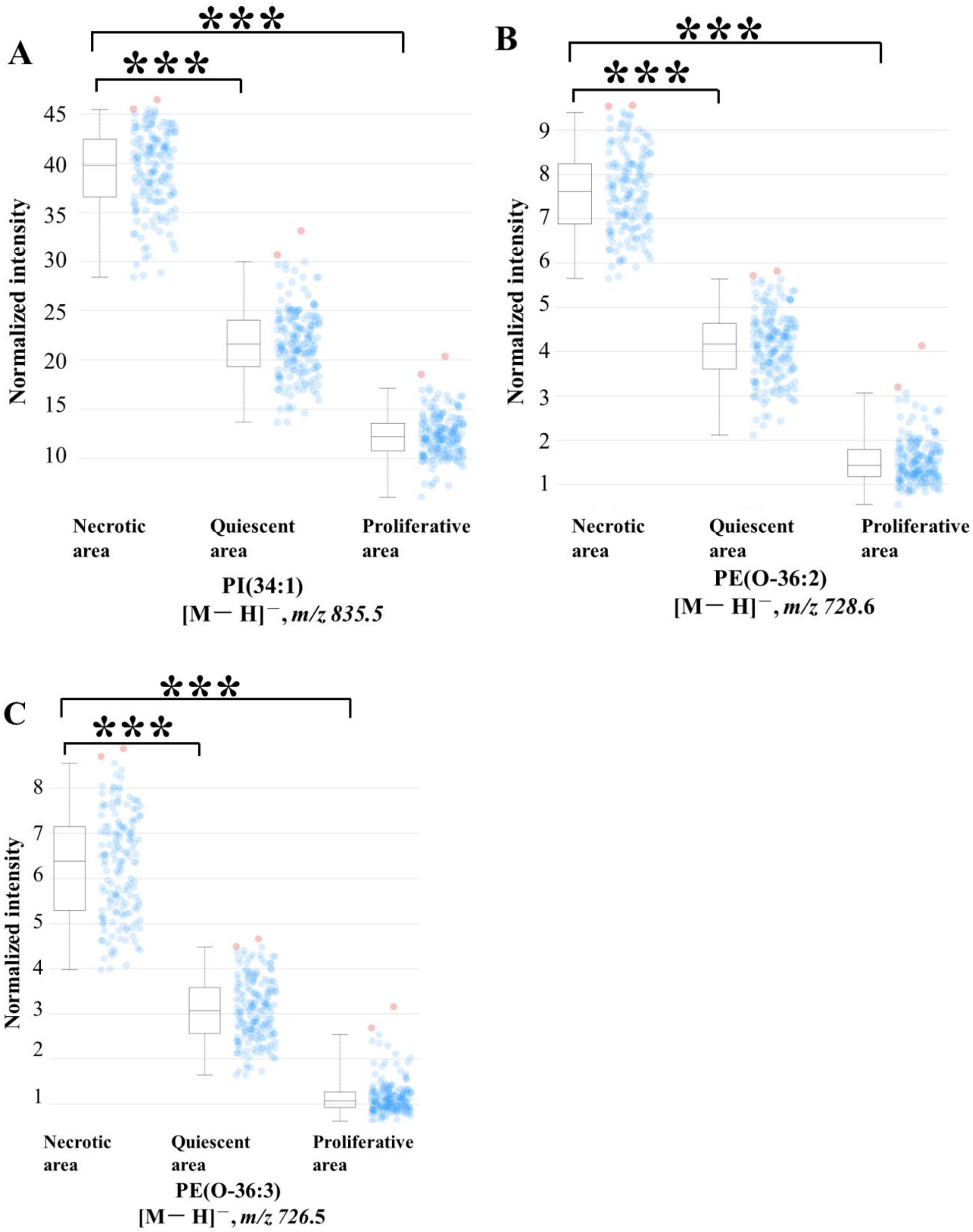

Figure S5. Statistical analysis of intensities of three different regions in cell spheroids (n = 3). A) PI(34:1). B) PE(O-36:2). C) PE(O-36:3). Red dots represented outliers. *$p < 0.05$, **$p < 0.01$, ***$p < 0.001$.

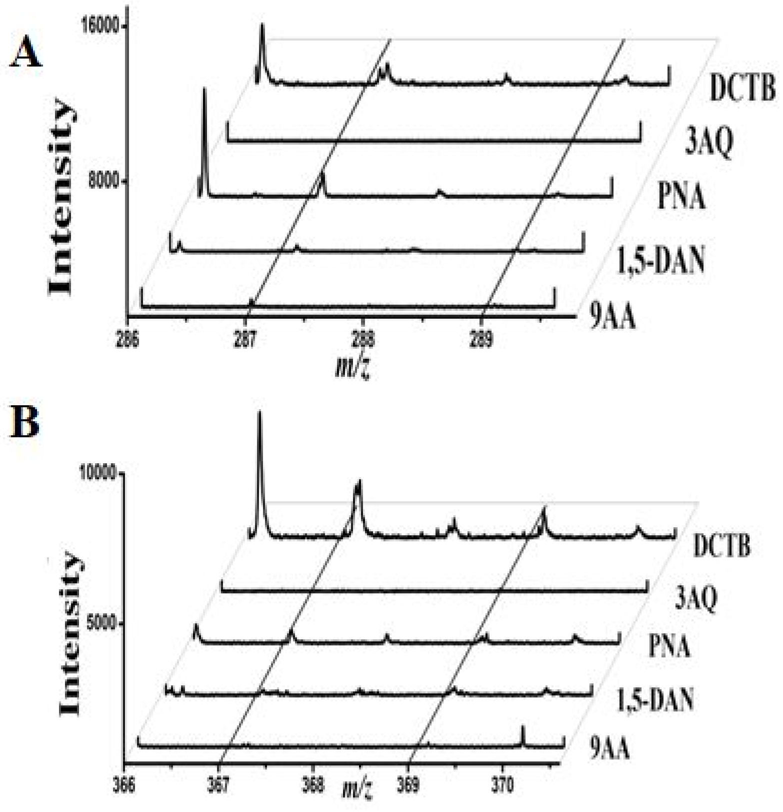

Figure S6. MALDI spectra of five matrices. The range of mass spectra were within (A) *m/z* 286.0-289.5 and (B) *m/z* 366.0-370.5. Different solution of matrices were pipetted onto a stainless-steel plate and mass spectra were acquired by an instrument named rapiflex MALDI-TOF Tissuetyper.The instrument was calibrated by two ions (*m/z* 223.062 and *m/z* 447.130) of matrix of sinapic acid in linear mode.

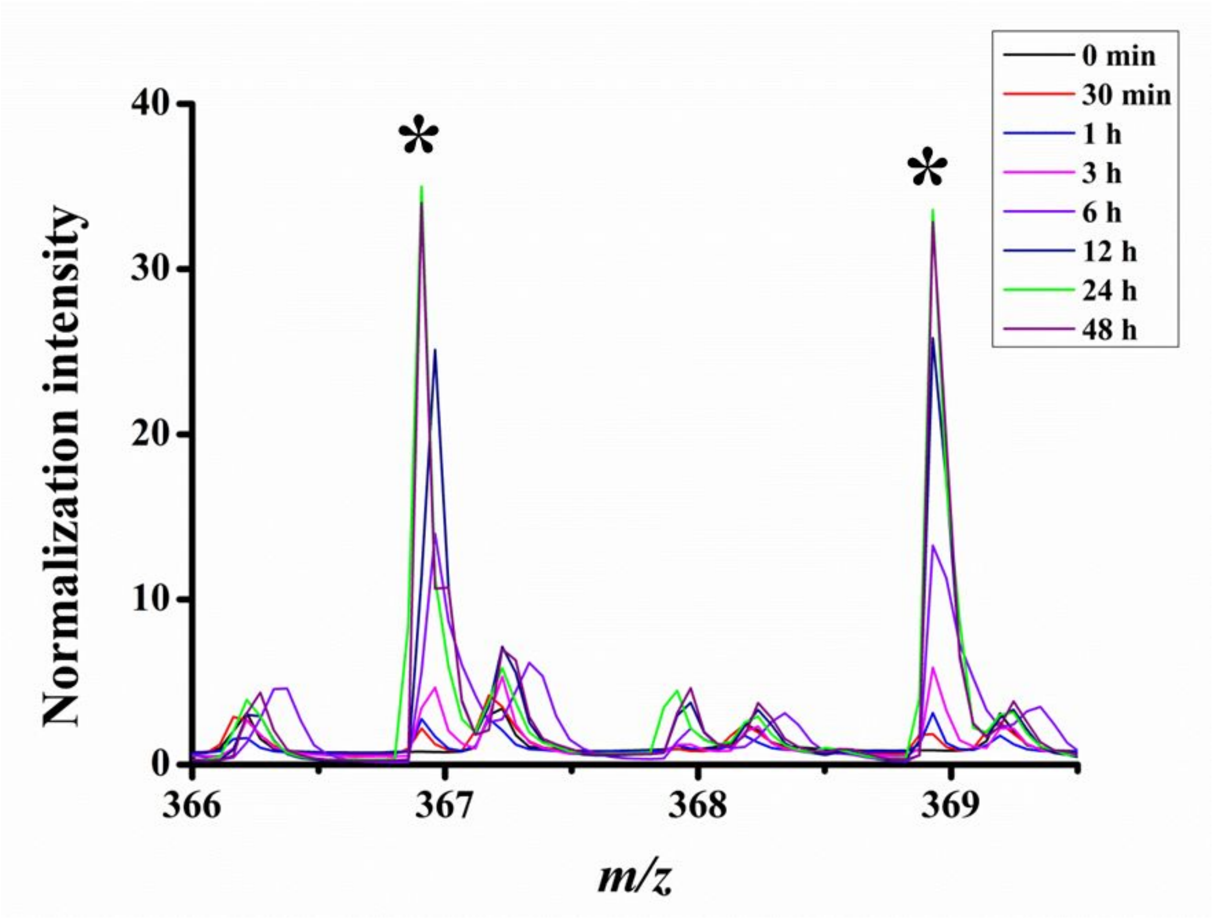

Figure S7. Representative MALDI-MS spectra of TCSS in CCS treated with TCS (10 μM) at different time points. Spectra of the middle sections of CCS treated with TCS at different time points were acquired and normalized by the total ion count. Asterisks indicated detected ion peaks of TCSS.

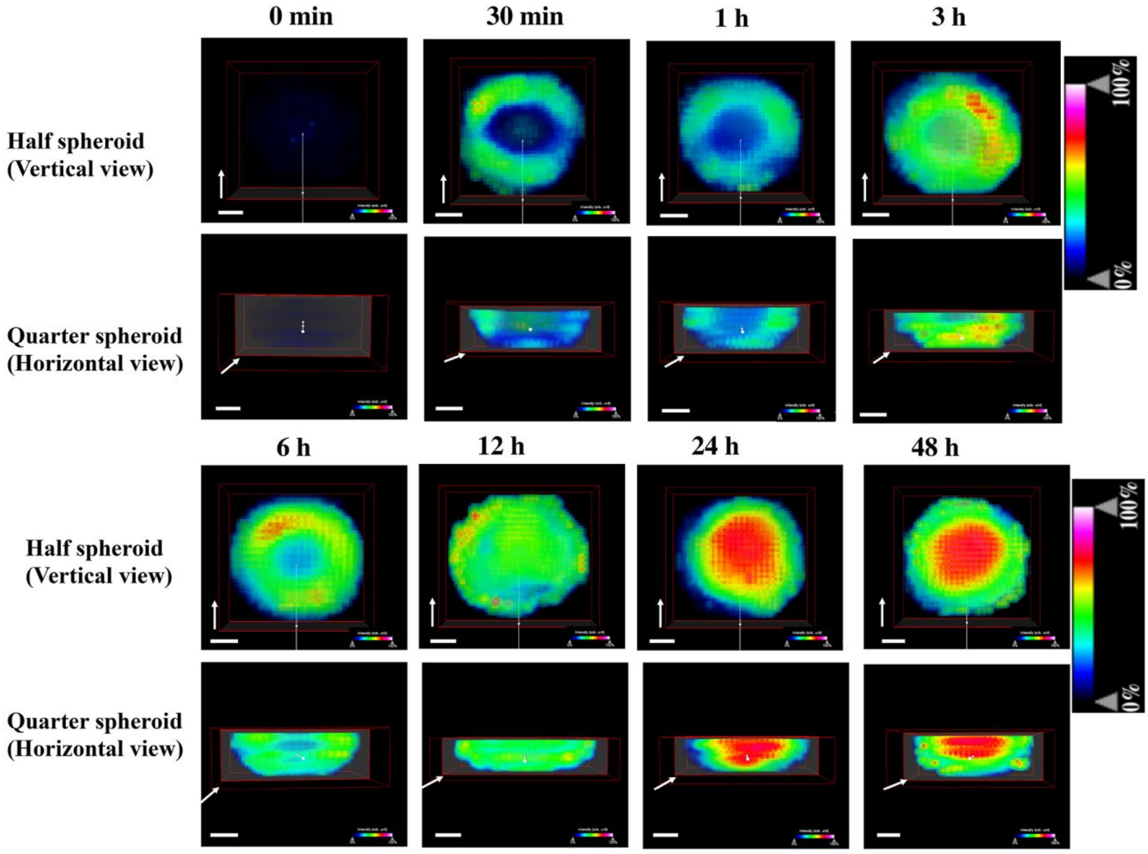

.

Figure S8. The distribution of TCSS ion (*m/z* 368.9) in CCS at different time points. The scale bars in all 3D ion images were 200 μm. The direction of the white arrows indicated the moving direction of the clipping planes.

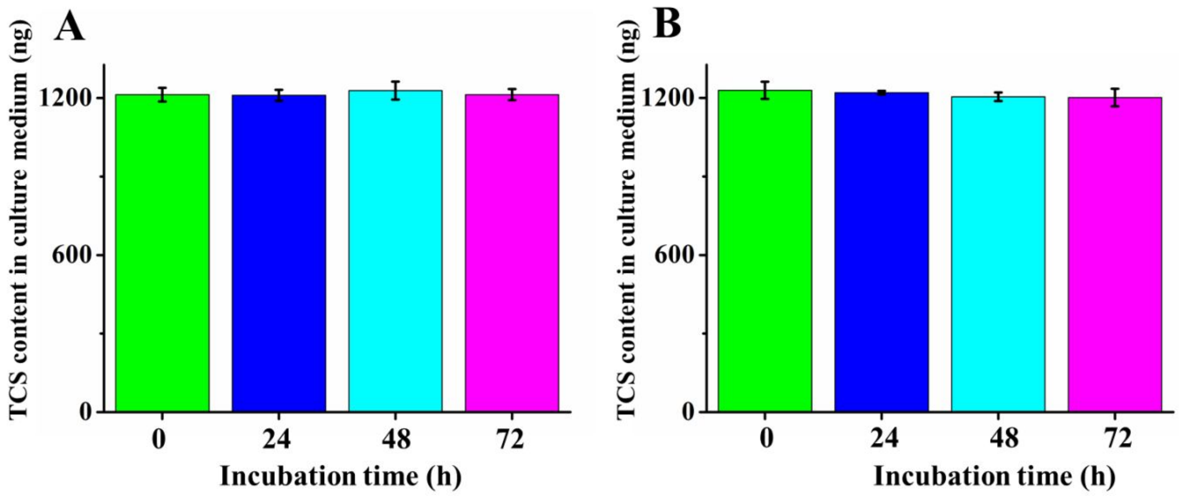

Figure S9. TCS content in culture medium. (A) TCS content in culture medium without cancer cell spheroids. (B) TCS content in culture medium with cancer cell spheroids irradiated by UV light. Each time point contained three sample replicates. Each sample contained 1 mL of culture medium. The error bars represented SD. The statistical analysis was performed between adjacent time points. *$p < 0.05$, **$p < 0.01$, ***$p < 0.001$.

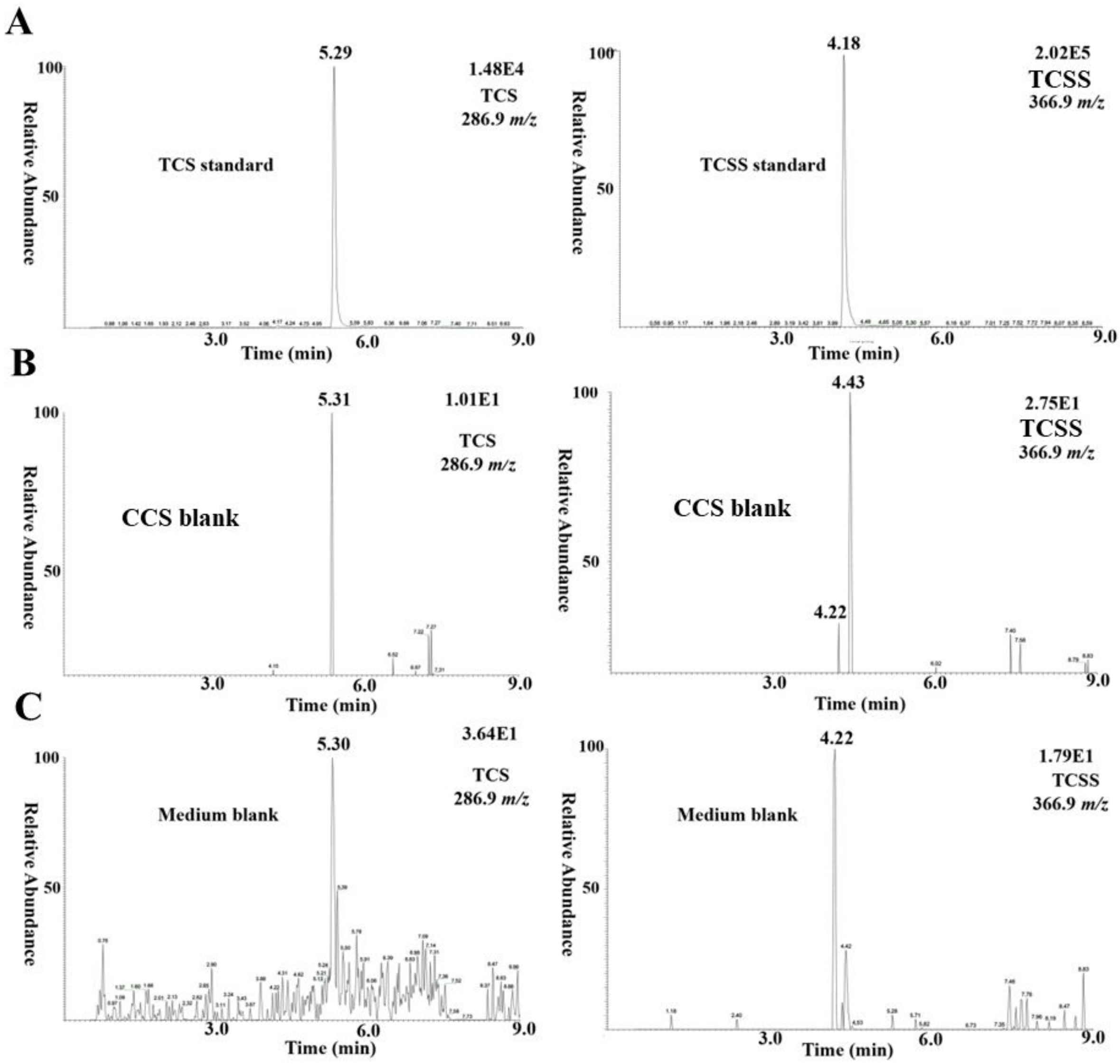

Figure S10. Mass spectra of TCS and TCSS in samples of (A) standards, (B) CCS blank and (C) medium blank. Mass spectra were acquired by a UPLC system coupled to a TSQ Quantiva Triple Quadrupole Mass Spectrometer.

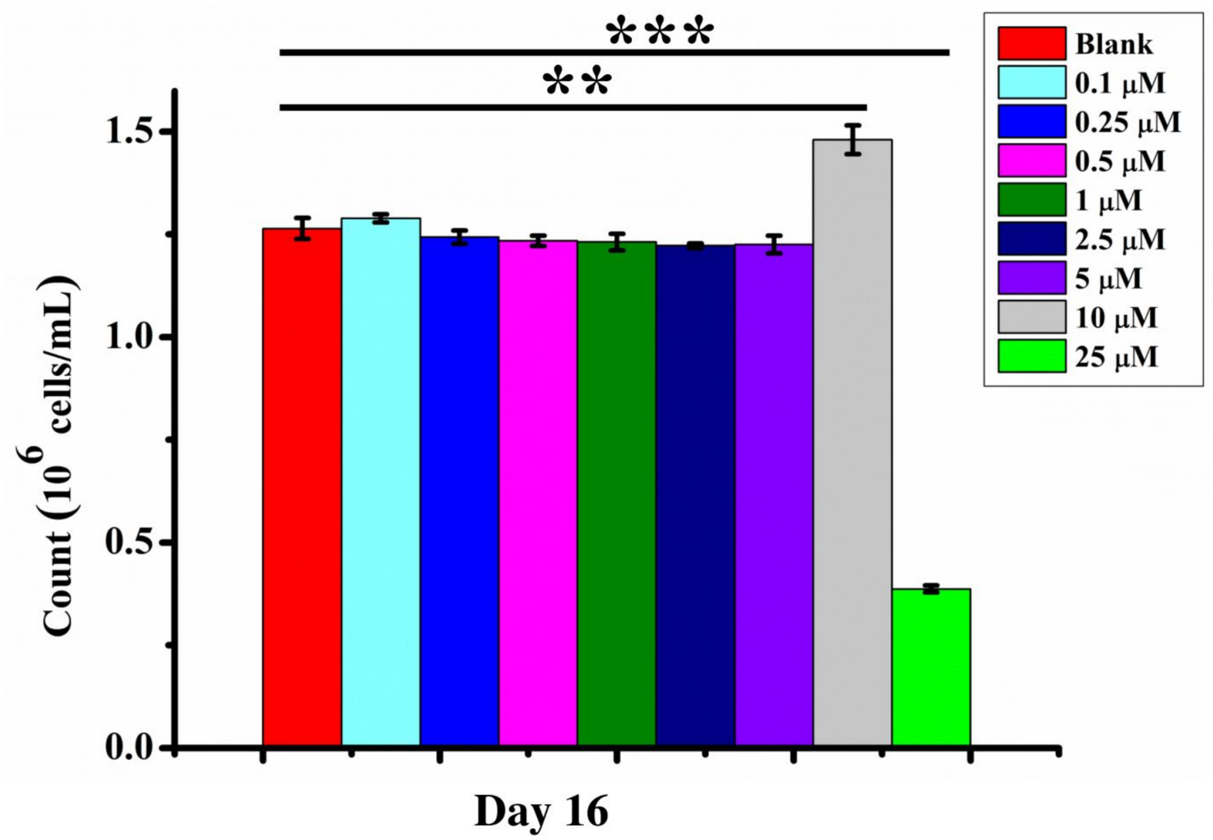

Figure S11. Cell numbers of HCT116 CCS exposed to various concentrations of TCS on day 16 in culture (n = 5). One Cell spheroid in each well was digested by 0.25% trypsin (100 μL) for 60 min. The cell number of each cell spheroid was counted by using an automated cell counter.

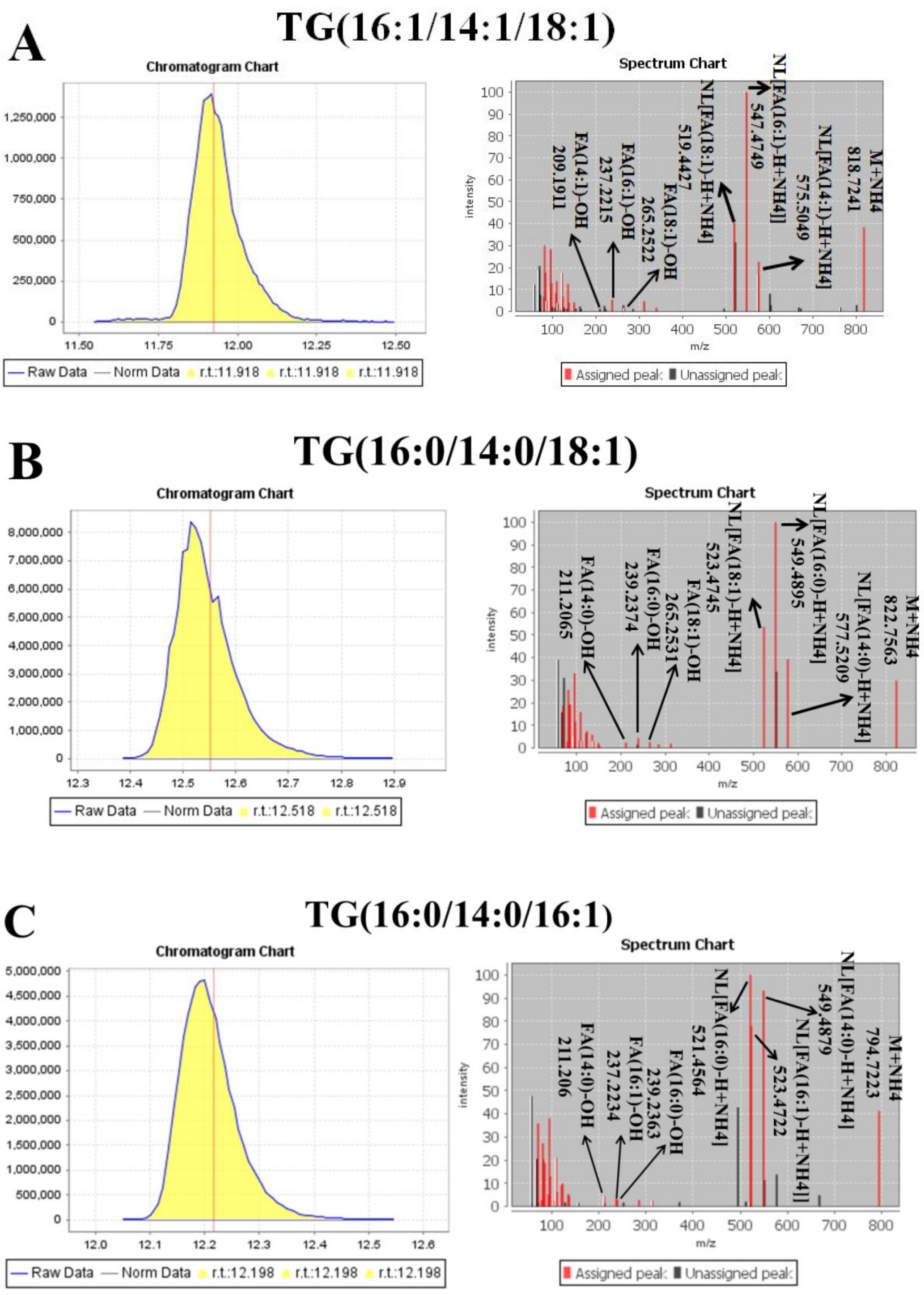

Figure S12. The chromatogram and MS/MS information of lipid species that were significantly changed. (A) TG(16:1/14:1/18:1). (B) TG(16:0/14:0/18:1). (C) TG(16:0/14:0/16:1).

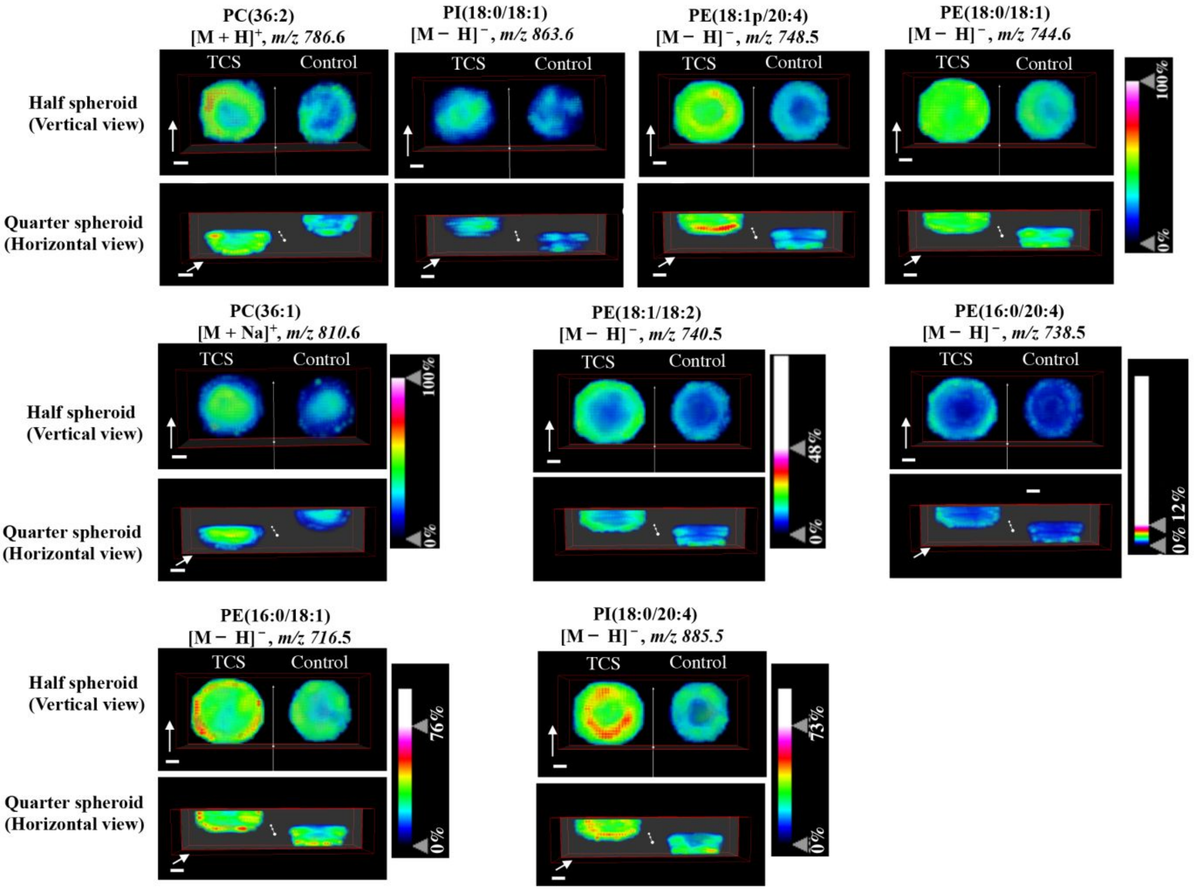

Figure S13. Ion images of lipids in the control and TCS-treated groups. The scale bars in all 3D ion images were 200 μm. The direction of the white arrows indicated the moving direction of the clipping planes.

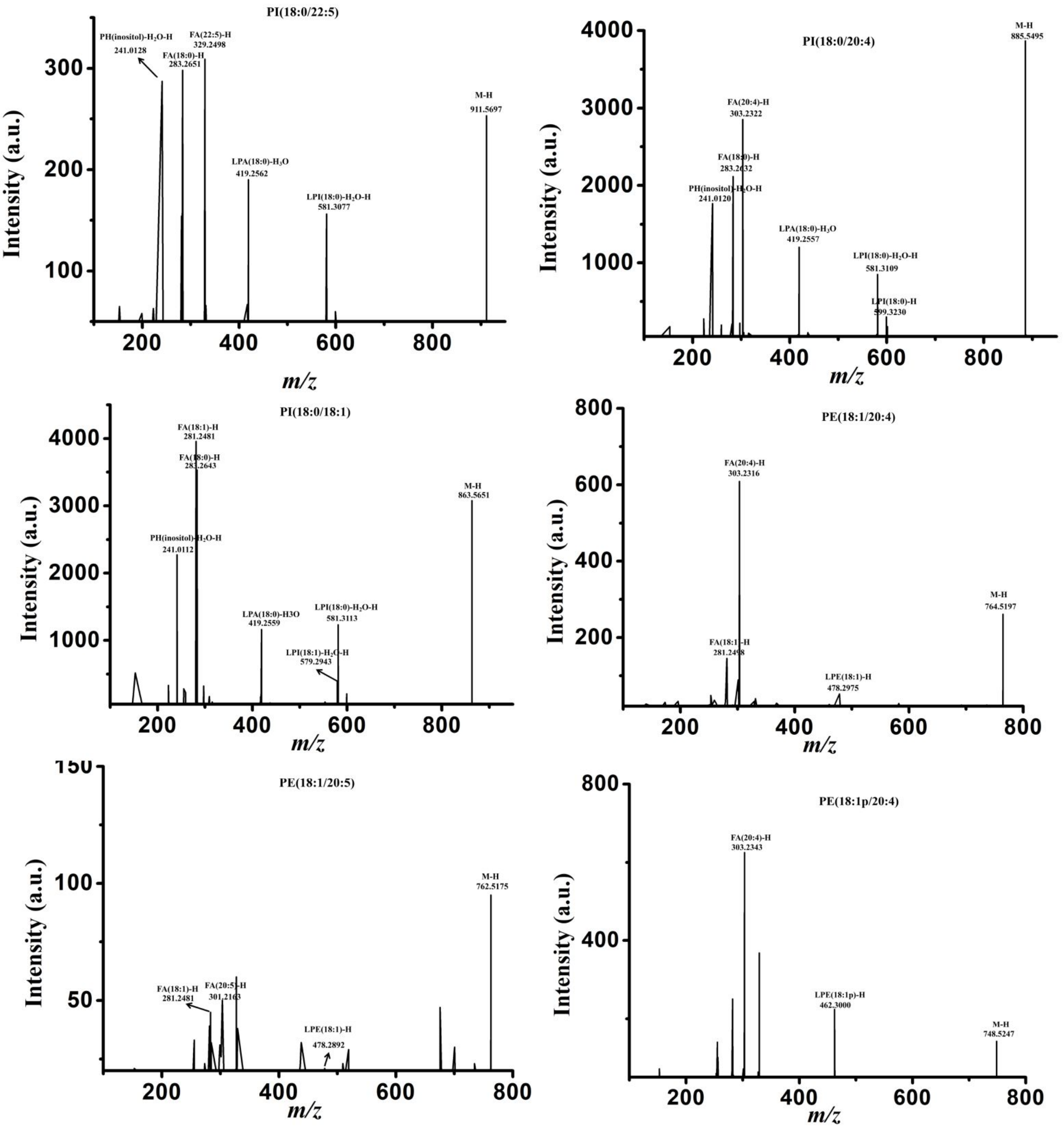
PI(18:0/22:5)
PH(inositol)-H2O-H
241.0128
FA(22:5)-H
329.2498
FA(18:0)-H
283.2651
M-H
911.5697
LPA(18:0)-H3O
419.2562
LPI(18:0)-H2O-H
581.3077
Intensity (a.u.)
m/z
PI(18:0/20:4)
M-H
885.5495
FA(20:4)-H
303.2322
FA(18:0)-H
283.2632
PH(inositol)-H2O-H
241.0120
LPA(18:0)-H3O
419.2557
LPI(18:0)-H2O-H
581.3109
LPI(18:0)-H
599.3230
PI(18:0/18:1)
FA(18:1)-H
281.2481
FA(18:0)-H
283.2643
M-H
863.5651
PH(inositol)-H2O-H
241.0112
LPA(18:0)-H3O
419.2559
LPI(18:0)-H2O-H
581.3113
LPI(18:1)-H2O-H
579.2943
PE(18:1/20:4)
FA(20:4)-H
303.2316
M-H
764.5197
FA(18:1)-H
281.2498
LPE(18:1)-H
478.2975
PE(18:1/20:5)
M-H
762.5175
FA(18:1)-H
281.2481
FA(20:5)-H
301.2163
LPE(18:1)-H
478.2892
PE(18:1p/20:4)
FA(20:4)-H
303.2343
LPE(18:1p)-H
462.3000
M-H
748.5247

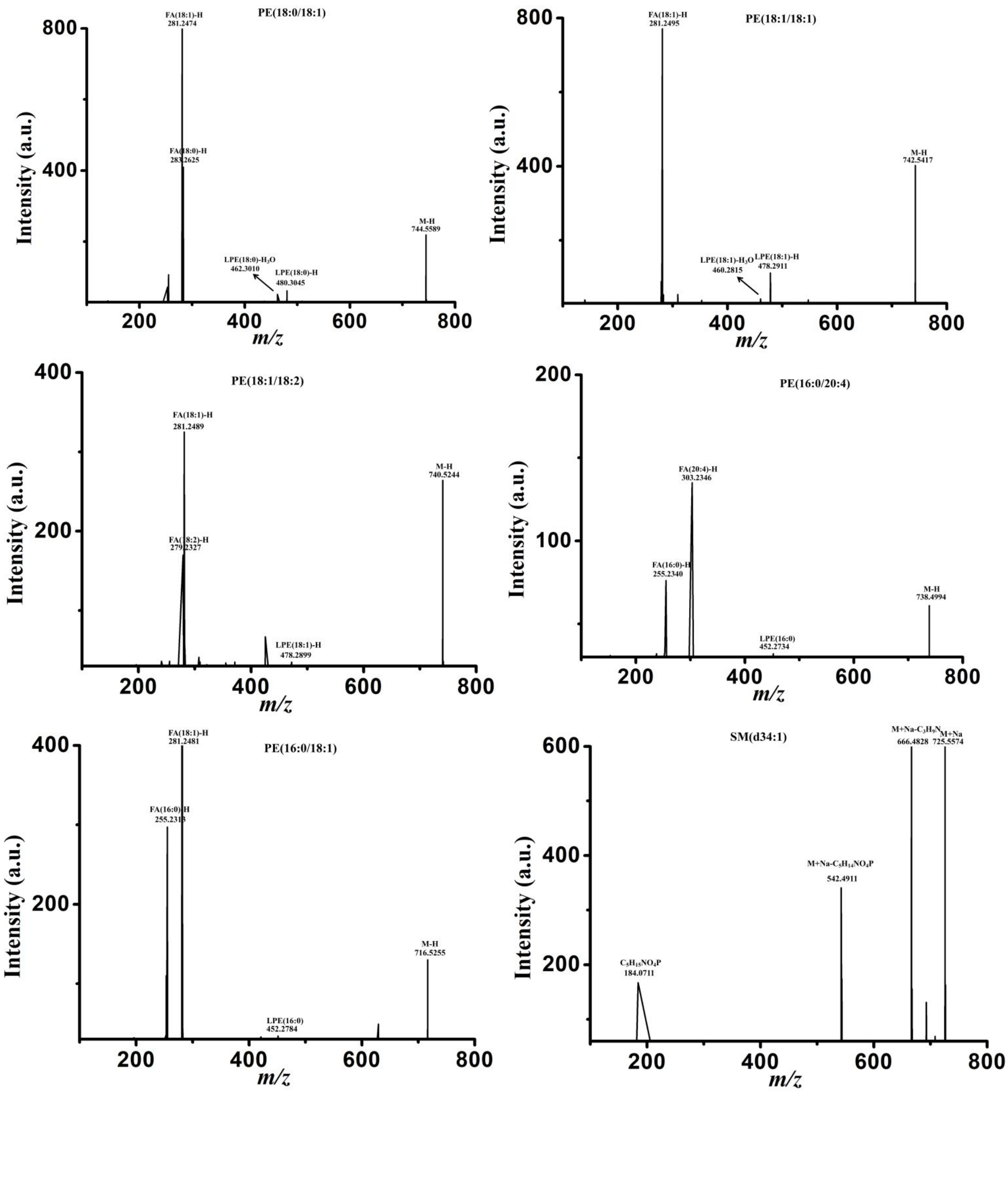
PE(18:0/18:1)
FA(18:1)-H
281.2474
FA(18:0)-H
283.2625
LPE(18:0)-H_2O
462.3010
LPE(18:0)-H
480.3045
M-H
744.5589
PE(18:1/18:1)
FA(18:1)-H
281.2495
LPE(18:1)-H_2O
460.2815
LPE(18:1)-H
478.2911
M-H
742.5417
PE(18:1/18:2)
FA(18:1)-H
281.2489
FA(18:2)-H
279.2327
LPE(18:1)-H
478.2899
M-H
740.5244
PE(16:0/20:4)
FA(20:4)-H
303.2346
FA(16:0)-H
255.2340
LPE(16:0)
452.2734
M-H
738.4994
PE(16:0/18:1)
FA(18:1)-H
281.2481
FA(16:0)-H
255.2313
LPE(16:0)
452.2784
M-H
716.5255
SM(d34:1)
M+Na-C_3H_9N
666.4828
M+Na
725.5574
M+Na-$C_5H_{14}NO_4P$
542.4911
$C_5H_{15}NO_4P$
184.0711
Intensity (a.u.)
m/z

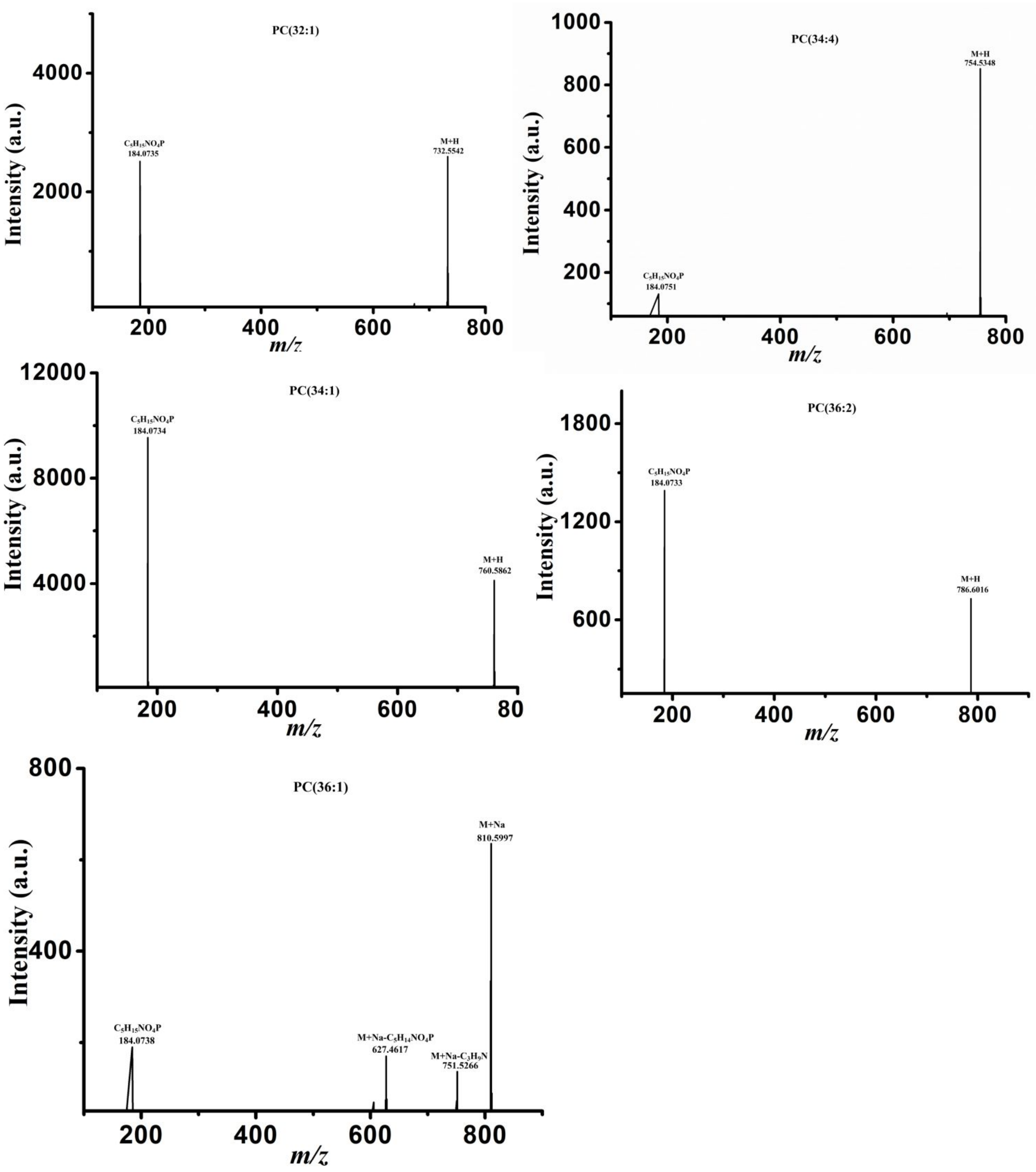

Figure S14. Lipids identified by MALDI-MS/MS using the timsTOF flex MALDI-2 instrument.

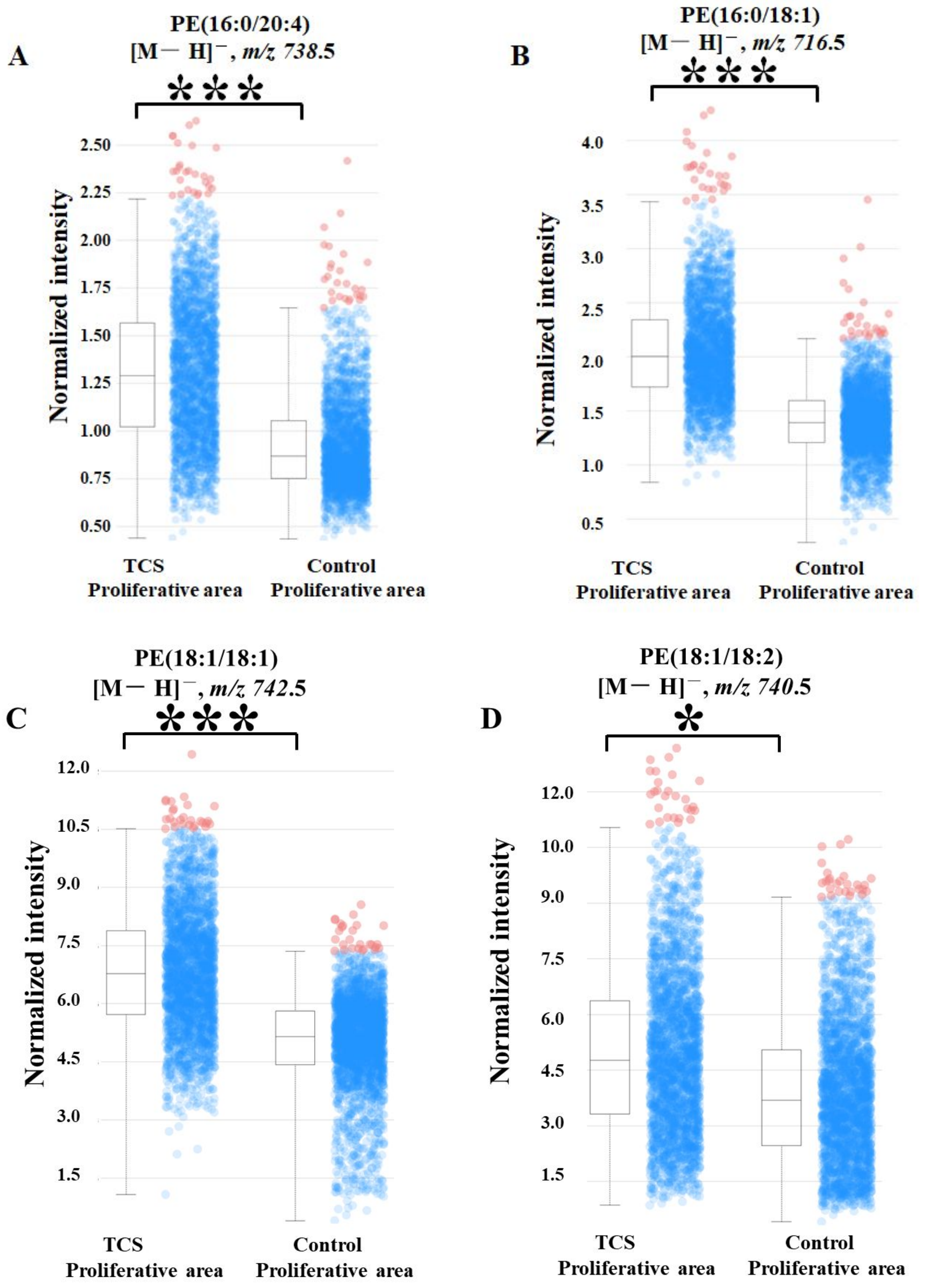

A
PE(16:0/20:4)
[M− H]⁻, m/z 738.5
***
Normalized intensity
2.50
2.25
2.00
1.75
1.50
1.25
1.00
0.75
0.50
TCS
Proliferative area
Control
Proliferative area
B
PE(16:0/18:1)
[M− H]⁻, m/z 716.5
***
Normalized intensity
4.0
3.5
3.0
2.5
2.0
1.5
1.0
0.5
TCS
Proliferative area
Control
Proliferative area
C
PE(18:1/18:1)
[M− H]⁻, m/z 742.5
***
Normalized intensity
12.0
10.5
9.0
7.5
6.0
4.5
3.0
1.5
TCS
Proliferative area
Control
Proliferative area
D
PE(18:1/18:2)
[M− H]⁻, m/z 740.5
*
Normalized intensity
12.0
10.0
9.0
7.5
6.0
4.5
3.0
1.5
TCS
Proliferative area
Control
Proliferative area

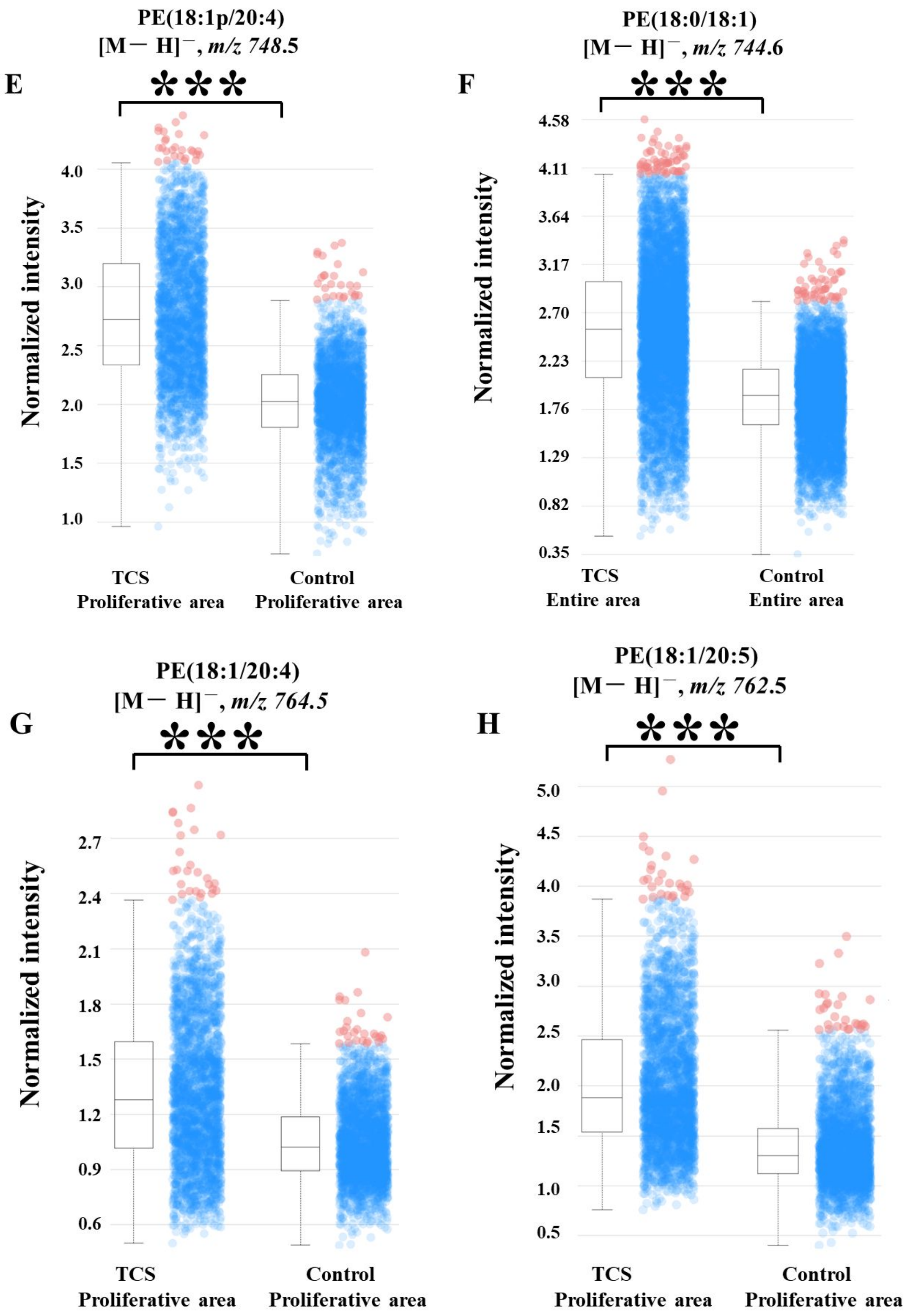
E
PE(18:1p/20:4)
[M− H]⁻, m/z 748.5
***
Normalized intensity
4.0
3.5
3.0
2.5
2.0
1.5
1.0
TCS
Proliferative area
Control
Proliferative area
F
PE(18:0/18:1)
[M− H]⁻, m/z 744.6
***
Normalized intensity
4.58
4.11
3.64
3.17
2.70
2.23
1.76
1.29
0.82
0.35
TCS
Entire area
Control
Entire area
G
PE(18:1/20:4)
[M− H]⁻, m/z 764.5
***
Normalized intensity
2.7
2.4
2.1
1.8
1.5
1.2
0.9
0.6
TCS
Proliferative area
Control
Proliferative area
H
PE(18:1/20:5)
[M− H]⁻, m/z 762.5
***
Normalized intensity
5.0
4.5
4.0
3.5
3.0
2.5
2.0
1.5
1.0
0.5
TCS
Proliferative area
Control
Proliferative area

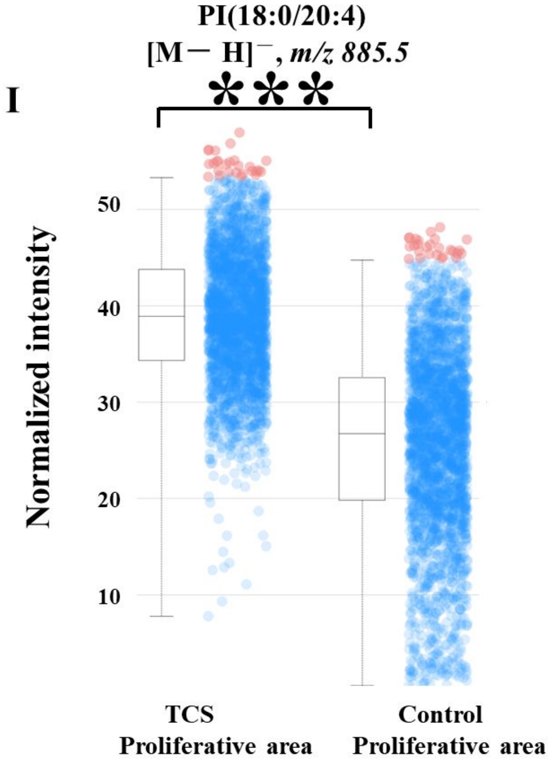
PI(18:0/20:4)
[M− H]⁻, m/z 885.5
I
***
Normalized intensity
50
40
30
20
10
TCS
Proliferative area
Control
Proliferative area

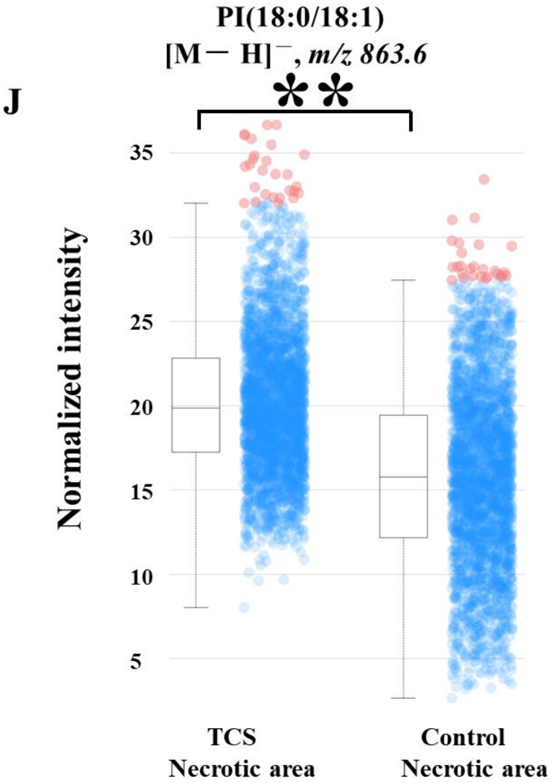
PI(18:0/18:1)
[M− H]⁻, m/z 863.6
J
**
Normalized intensity
35
30
25
20
15
10
5
TCS
Necrotic area
Control
Necrotic area

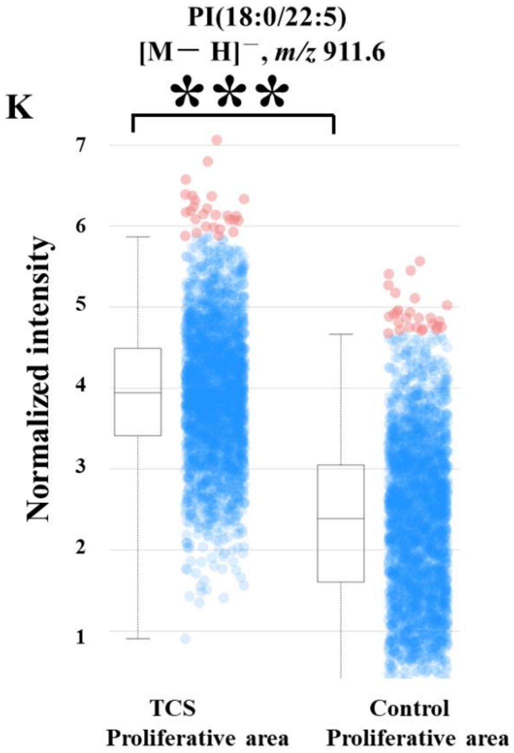
PI(18:0/22:5)
[M− H]⁻, m/z 911.6
K
***
Normalized intensity
7
6
5
4
3
2
1
TCS
Proliferative area
Control
Proliferative area

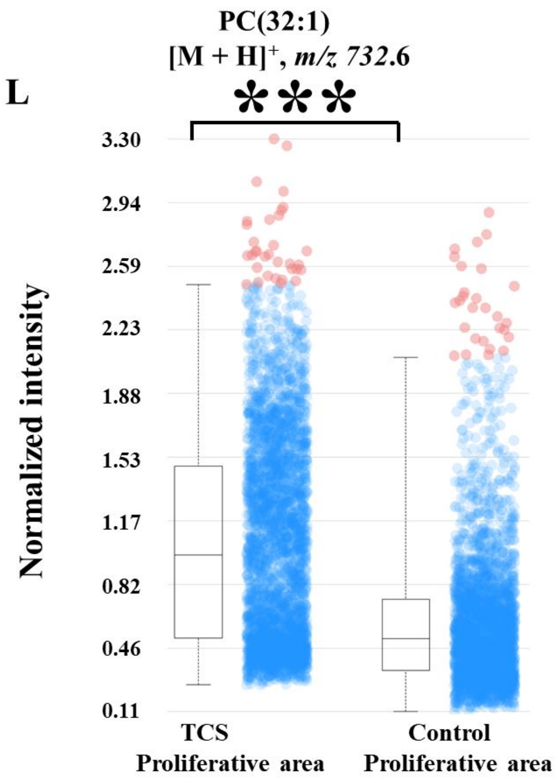
PC(32:1)
[M + H]+, m/z 732.6
L
***
3.30
2.94
2.59
2.23
1.88
1.53
1.17
0.82
0.46
0.11
Normalized intensity
TCS
Proliferative area
Control
Proliferative area

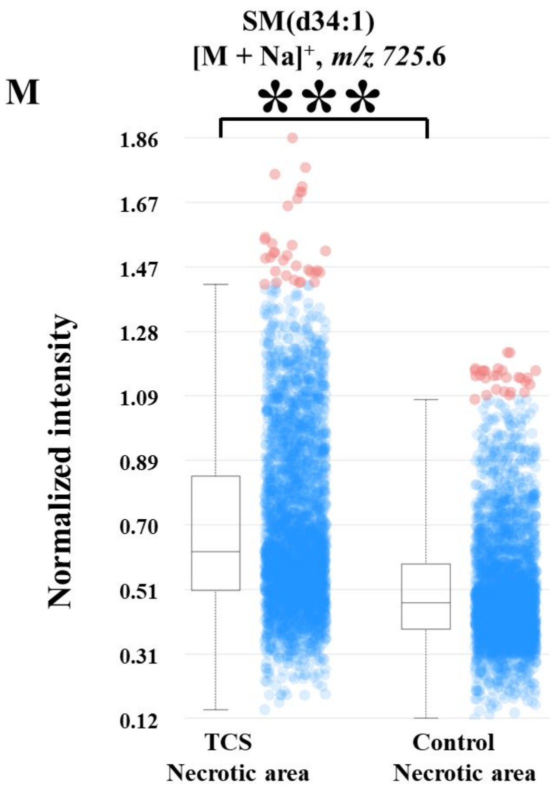
SM(d34:1)
[M + Na]+, m/z 725.6
M
***
1.86
1.67
1.47
1.28
1.09
0.89
0.70
0.51
0.31
0.12
Normalized intensity
TCS
Necrotic area
Control
Necrotic area

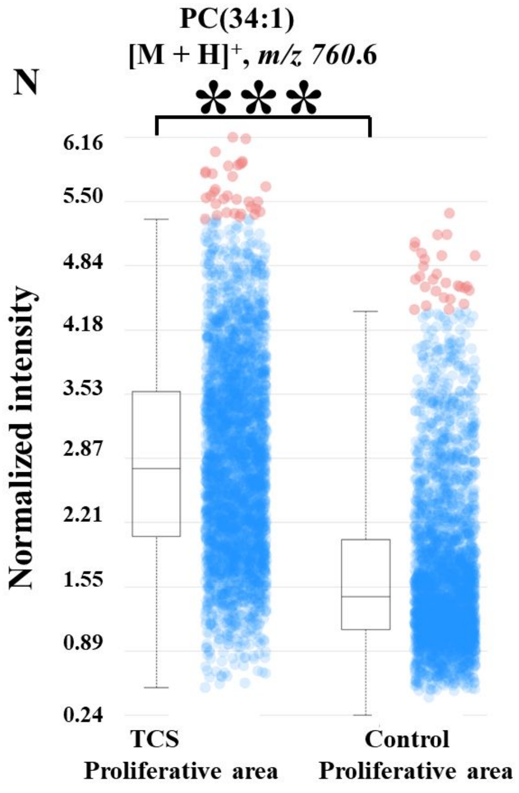
PC(34:1)
[M + H]+, m/z 760.6
N
***
6.16
5.50
4.84
4.18
3.53
2.87
2.21
1.55
0.89
0.24
Normalized intensity
TCS
Proliferative area
Control
Proliferative area

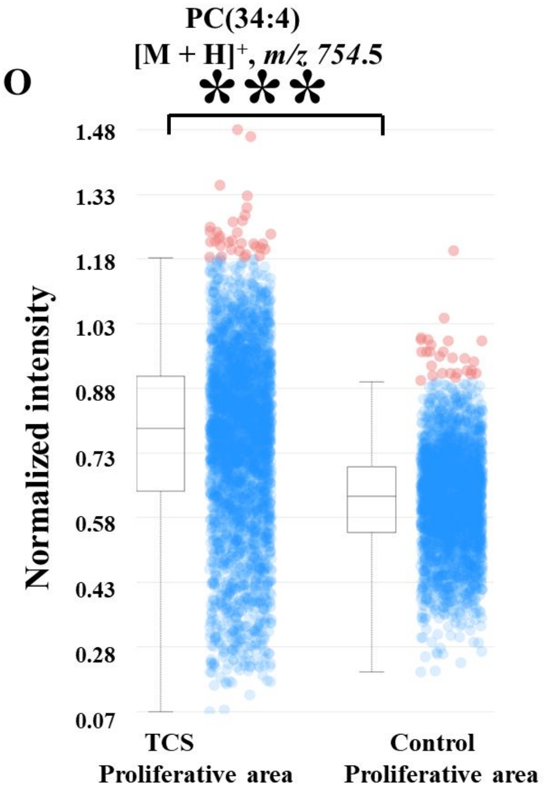
PC(34:4)
[M + H]+, m/z 754.5
O
***
1.48
1.33
1.18
1.03
0.88
0.73
0.58
0.43
0.28
0.07
Normalized intensity
TCS
Proliferative area
Control
Proliferative area

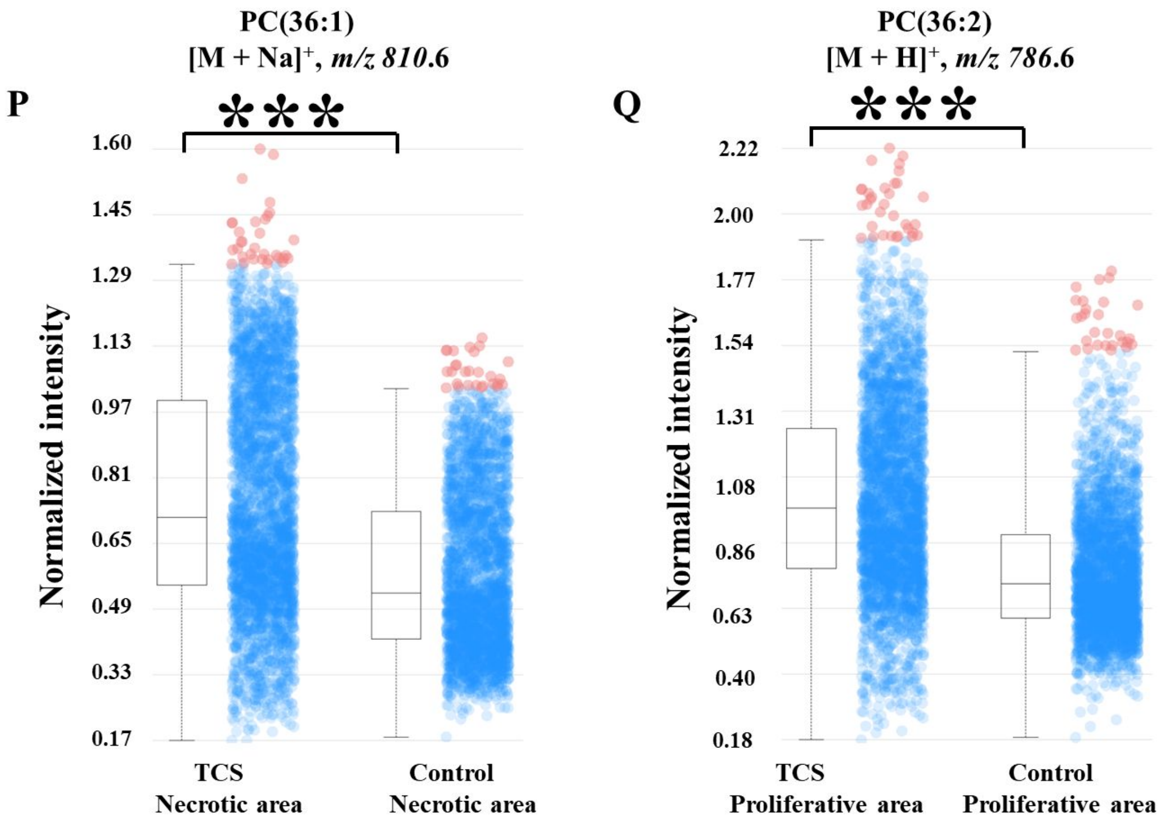

Figure S15. Statistical analysis of intensities of different lipids between control and TCS-treated groups (n=15). A) PE(16:0/20:4). B) PE(16:0/18:1). C) PE(18:1/18:1). D) PE(18:1/18:2). E) PE(18:1p/20:4). F) PE(18:0/18:1). G) PE(18:1/20:4). H) PE(18:1/20:5). I) PI(18:0/20:4). J) PI(18:0/18:1). K) PI(18:0/22:5). L) PC(32:1). M) SM(d34:1). N) PC(34:1). O) PC(34:4) P) PC(36:1). Q) PC(36:2). Red dots represented outliers. *$p < 0.05$, **$p < 0.01$, ***$p < 0.001$.

Table S1 Information of statistical analysis of TCS and TCSS in cell spheroids and culture medium at different exposure time. Each time points contained five biological replicates.

No	Name	Group	Fold change (High time/Low time, mean ± SD)	*p* value
1	TCS content in cell spheroids			
		0.5 h & 1h	1.81 ± 0.25	0.0005
		1h & 3h	2.18 ± 0.29	0.0002
		3h & 6h	1.88 ± 0.21	<0.0001
		6h & 12h	1.35 ± 0.17	0.0050
		12h & 24h	0.65 ± 0.06	0.0005
		24h & 48h	0.73 ± 0.48	0.2448
		48h & 72h	0.57 ± 0.16	0.2001
2	TCSS content in cell spheroids			
		0.5 h & 1h	1.34 ± 0.26	0.0903
		1h & 3h	4.95 ± 0.87	0.0004
		3h & 6h	1.78 ± 0.28	0.0009

		6h & 12h	1.35 ± 0.18	<0.0001
		12h & 24h	1.31 ± 0.21	0.0233
		24h & 48h	0.99 ± 0.17	0.9161
		48h & 72h	1.03 ± 0.42	0.8793
3	TCS content in culture medium			
		0.5 h & 1h	1.00 ± 0.01	0.6882
		1h & 3h	1.01 ± 0.02	0.5857
		3h & 6h	0.97 ± 0.03	0.1346
		6h & 12h	1.02 ± 0.01	0.2054
		12h & 24h	0.83 ± 0.04	<0.0001
		24h & 48h	0.76 ± 0.03	<0.0001
		48h & 72h	0.59 ± 0.03	<0.0001
4	TCSS content in culture medium			
		0.5 h & 1h	5.05 ± 0.88	<0.0001
		1h & 3h	6.19 ± 0.31	<0.0001
		3h & 6h	3.77 ± 0.13	<0.0001

6h & 12h	1.65 ± 0.06	<0.0001
12h & 24h	1.75 ± 0.02	<0.0001
24h & 48h	1.96 ± 0.04	<0.0001
48h & 72h	1.39 ± 0.02	<0.0001

Table S2 Information of lipid markers identified by UPLC-MS/MS. The letter "P" and "N" refer to the "positive" and "negative", respectively. For lipid species that were detected in both positive and negative ionization modes, their fold changes and p-values in either negative ionization mode or positive ionization mode were selected. For lipid species in PE, PI and LPE, fold changes and p-values in negative ionization modes were selected. For PC and SM, fold changes and p-values in negative ionization mode were selected. Each group contained eight biological replicates.

No	Name	Retention time (min)	Detection mode	Fold change (Exp/Con, mean ± SD)	*p* value
1	TG(16:0e/16:0/18:0)	13.76	P	1.93 ± 0.36	0.0001
2	TG(18:0e/14:0/16:0)	13.40	P	1.68 ± 0.27	<0.0001
3	TG(16:1/17:1/18:1)	12.35	P	1.67 ± 0.43	<0.0022
4	TG(18:1/12:0/14:0)	11.88	P	1.46 ± 0.30	0.0031
5	TG(16:0/18:1/24:0)	14.26	P	1.43 ± 0.35	0.0248
6	TG(16:0/16:0/18:1)	12.86	P	1.36 ± 0.24	0.0022
7	TG(16:1/14:0/17:1)	12.05	P	1.36 ± 0.21	0.0020
8	TG(16:1/18:1/18:2)	12.25	P	1.35 ± 0.30	0.0131
9	TG(18:0p/14:0/16:0)	13.02	P	1.35 ± 0.22	0.0014

10	TG(16:0/14:0/16:0)	12.55	P	1.34 ± 0.28	0.0198
11	TG(16:0p/14:0/16:0)	12.68	P	1.32 ± 0.25	0.0123
12	TG(16:0/18:1/20:4)	12.42	P	1.31 ± 0.21	0.0034
13	TG(20:1/18:1/22:6)	12.41	P	1.31 ± 0.23	0.0062
14	TG(16:1/12:0/18:1)	11.88	P	1.31 ± 0.22	0.0087
15	TG(16:1/18:1/18:1)	12.50	P	1.31 ± 0.21	0.0048
16	TG(16:0/16:1/17:1)	12.35	P	1.31 ± 0.22	0.0051
17	TG(18:0p/16:1/18:1)	13.00	P	1.30 ± 0.24	0.0110
18	TG(20:1p/16:0/18:1)	13.32	P	1.30 ± 0.24	0.0258
19	TG(16:1/12:0/14:0)	11.57	P	1.29 ± 0.29	0.0268
20	TG(16:0/14:0/16:1)	12.19	P	1.29 ± 0.26	0.0414
21	TG(18:1/18:1/20:4)	12.40	P	1.28 ± 0.23	0.0091
22	TG(16:0/16:1/18:1)	12.50	P	1.28 ± 0.21	0.0088
23	TG(16:0/14:0/18:1)	12.52	P	1.28 ± 0.18	0.0083
24	TG(16:1/14:1/18:1)	11.92	P	1.28 ± 0.21	0.0140
25	TG(15:0/14:0/16:1)	12.04	P	1.26 ± 0.26	0.0326
26	TG(16:1/16:1/18:1)	12.21	P	1.25 ± 0.19	0.0177

27	TG(16:0/18:1/18:1)	12.84	P	1.25 ± 0.19	0.0237
28	TG(18:1/18:1/18:1)	12.80	P	1.25 ± 0.21	0.0310
29	TG(16:1/14:0/18:1)	12.20	P	1.25 ± 0.22	0.0326
30	TG(16:0/18:1/22:6)	12.13	P	1.25 ± 0.20	0.0201
31	TG(15:0/16:0/16:0)	12.71	P	1.23 ± 0.24	0.0328
32	TG(18:0p/16:0/18:1)	13.34	P	1.22 ± 0.22	0.0470
33	TG(16:0/17:0/18:1)	13.03	P	0.79 ± 0.15	0.0134
34	TG(26:0/16:0/18:1)	14.61	P	0.75 ± 0.12	0.0012
35	TG(18:1/22:1/22:5)	13.02	P	0.75 ± 0.11	0.0015
36	TG(19:1/18:1/20:3)	12.76	P	0.70 ± 0.12	0.0007
37	TG(18:1/18:1/22:2)	13.18	P	0.66 ± 0.10	0.0005
38	TG(18:0/18:1/20:3)	12.92	P	0.63 ± 0.11	0.0002
39	TG(15:0/16:0/18:1)	12.69	P	0.60 ± 0.18	0.0023
40	SM(d34:0)	8.90	P	1.33 ± 0.21	0.002
41	SM(d44:4)	10.59	P	1.32 ± 0.23	0.0060
42	SM(d32:1)	7.76	P	1.29 ± 0.24	0.0099
43	SM(d42:1+pO)	10.15	P	1.22 ± 0.18	0.0103

44	SM(d42:2)	10.41	P	1.25 ± 0.21	0.0111
45	SM(d42:1)	10.85	P	1.25 ± 0.20	0.0198
46	SM(d34:2)	7.85	P	1.23 ± 0.19	0.0120
47	SM(d40:1)	10.46	P + N	1.30 ± 0.22	0.0057
48	SM(d38:2)	9.42	P	1.29 ± 0.24	0.0155
49	SM(d38:1)	9.98	P + N	1.22 ± 0.20	0.0247
50	SM(d36:4)	8.56	P	1.37 ± 0.24	0.0032
51	SM(d44:3)	10.43	P	1.26 ± 0.27	0.0337
52	SM(d35:3+pO)	9.84	P	1.32 ± 0.23	0.0056
53	SM(d34:1)	8.58	P	1.21 ± 0.19	0.0143
54	SM(d18:2/18:1)	8.90	H	1.32 ± 0.35	0.034
55	PI(18:0/22:5)	8.76	P + N	1.25 ± 0.20	0.0130
56	PI(18:0/20:4)	8.77	P + N	1.21 ± 0.18	0.0135
57	PI(18:0/18:1)	9.42	P + N	1.31 ± 0.18	0.0010
58	PI(16:0/18:1)	8.72	P + N	1.25 ± 0.23	0.0185
59	PG(18:1/22:6)	7.60	N	1.21 ± 0.16	0.0078
60	PE(20:1/18:1)	10.07	P + N	1.35 ± 0.18	0.0012

61	PE(20:0p/22:5)	10.28	P	1.24 ± 0.23	0.0251
62	PE(20:0p/18:1)	10.78	P + N	1.24 ± 0.18	0.0079
63	PE(18:1p/22:6)	8.95	N	1.23 ± 0.22	0.0220
64	PE(18:1p/20:5)	8.70	P	1.57 ± 0.31	0.0009
65	PE(18:1p/20:4)	9.18	N	1.26 ± 0.22	0.0090
66	PE(18:1p/18:1)	9.84	P + N	1.23 ± 0.19	0.0159
67	PE(18:1/22:6)	8.64	N	1.41 ± 0.23	0.0008
68	PE(18:1/20:5)	8.36	N	1.78 ± 0.28	<0.0001
69	PE(18:1/20:4)	8.88	P + N	1.45 ± 0.23	0.0003
70	PE(18:1/20:3)	9.17	N	1.34 ± 0.19	0.0025
71	PE(18:1/18:2)	9.01	N	1.52 ± 0.24	0.0002
72	PE(18:1/18:1)	9.55	P + N	1.62 ± 0.17	<0.0001
73	PE(18:0p/18:1)	10.34	P	1.21 ± 0.16	0.0064
74	PE(18:0p/16:0)	10.33	P	1.53 ± 0.25	<0.0001
75	PE(18:0/20:4)	9.52	P + N	1.21 ± 0.19	0.0159
76	PE(18:0/18:1)	10.09	P + N	1.25 ± 0.17	0.0042
77	PE(16:1/18:1)	8.88	N	1.46 ± 0.25	0.0004

78	PE(16:0p/20:5)	8.69	P	1.53 ± 0.25	0.0001
79	PE(16:0p/20:4)	9.18	P + N	1.29 ± 0.20	0.0043
80	PE(16:0p/20:3)	9.47	P	1.29 ± 0.26	0.0144
81	PE(16:0p/16:0)	9.82	P	1.57 ± 0.24	0.0001
82	PE(16:0/20:4)	8.84	P + N	1.32 ± 0.22	0.0061
83	PE(16:0/18:1)	9.52	P+ N	1.28 ± 0.22	0.0077
84	PC(42:4p)	10.21	P	1.27 ± 0.25	0.0174
85	PC(40:8)	7.91	P	0.79 ± 0.11	0.0046
86	PC(40:7p)	8.77	P	0.61 ± 0.12	0.0001
87	PC(38:5)	8.69	N	1.56 ± 0.31	0.0002
88	PC(38:4)	9.34	P + N	1.21 ± 0.21	0.0247
89	PC(38:2)	9.91	P	1.24 ± 0.22	0.0231
90	PC(38:0e)	11.04	P	1.23 ± 0.21	0.0335
91	PC(36:4)	8.30	N	1.54 ± 0.29	0.0002
92	PC(36:2)	9.37	P + N	1.33 ± 0.23	0.0043
93	PC(36:1)	9.94	P	1.25 ± 0.21	0.0126
94	PC(36:0e)	10.68	P	1.25 ± 0.23	0.0202

95	PC(34:4)	7.88	P	1.27 ± 0.21	0.0070
96	PC(34:2)	8.70	P + N	1.30 ± 0.30	0.0033
97	PC(34:1)	9.34	P + N	1.23 ± 0.19	0.0097
98	PC(32:2)	8.02	P	1.21 ± 0.18	0.0069
99	PC(32:1)	8.69	P + N	1.44 ± 0.23	0.0007
100	PC(18:1/20:3)	8.98	N	1.30 ± 0.20	0.0130
101	PC(18:1/18:2)	8.82	N	1.50 ± 0.37	0.0132
102	PC(16:1/16:1)	9.37	P+N	1.23 ± 0.18	0.0087
103	PC(16:0p/22:5)	8.97	N	0.74 ± 0.16	0.0070
104	LPE(18:1)	3.26	N	1.26 ± 0.23	0.0187
105	LPE(18:0)	4.37	P + N	1.30 ± 0.18	0.0017
106	LPC(20:0)	5.36	P	1.22 ± 0.20	0.0173
107	LPC(18:0e)	4.74	P	1.22 ± 0.21	0.0191
108	LPC(18:0)	4.22	N	1.24 ± 0.20	0.0180
109	LPC(16:1)	2.09	N	1.68 ± 0.62	0.0200
110	DG(17:0/18:1)	10.59	P	1.23 ± 0.21	0.0331
111	DG(17:1/18:1)	10.14	P	1.27 ± 0.26	0.0275

112	DG(16:0/18:1)	10.36	P	1.30 ± 0.24	0.0073
113	DG(20:1/18:1)	10.78	P	1.30 ± 0.22	0.0123
114	DG(16:1/14:0)	9.21	P	1.30 ± 0.33	0.0328
115	DG(18:0/18:0)	11.17	P	1.31 ± 0.23	0.0053
116	DG(16:0/22:6)	9.56	P	1.31 ± 0.22	0.0099
117	DG(18:1/20:3)	10.08	P	1.31 ± 0.27	0.0203
118	DG(18:1/18:1)	10.35	P	1.32 ± 0.24	0.0083
119	DG(18:1/18:2)	9.97	P	1.33 ± 0.27	0.0141
120	DG(17:1/16:0)	10.11	P	1.33 ± 0.32	0.0218
121	DG(16:0/18:1)	10.35	P	1.34 ± 0.25	0.0056
122	DG(16:1/18:1)	9.88	P	1.34 ± 0.27	0.0090
123	DG(19:1/18:1)	10.57	P	1.36 ± 0.26	0.0042
124	DG(16:0/16:1)	9.84	P	1.37 ± 0.26	0.0044
125	DG(18:1/18:1)	10.36	P	1.37 ± 0.25	0.0037
126	DG(34:1e)	10.34	P	1.37 ± 0.26	0.0045
127	DG(34:2p)	10.33	P	1.38 ± 0.28	0.0037
128	DG(16:1/16:1)	9.25	P	1.40 ± 0.32	0.0089

129	DG(16:0/14:0)	9.83	P	1.40 ± 0.27	0.0021
130	DG(18:0/16:0)	10.80	P	1.42 ± 0.27	0.0014
131	DG(16:0/16:0)	10.35	P	1.47 ± 0.26	0.0005
132	DG(34:0p)	10.81	P	1.54 ± 0.33	0.0014
133	DG(32:1p)	10.32	P	1.56 ± 0.30	0.0007
134	Cer(d18:0/23:0)	11.24	P	0.75 ± 0.08	0.0015
135	Cer(d18:0/14:0)	8.91	P	0.74 ± 0.18	0.0041
136	Cer(d18:0/18:0)	10.21	P	0.76 ± 0.14	0.0171
137	Cer(d18:0/22:1)	10.65	P	0.70 ± 0.18	0.0013

Table S3 Information of lipid markers identified by MALDI MSI. The letter "P" and "N" refer to the "positive" and "negative", respectively. The number of biolgical replicates in each group was 15. Statistical comparison of 15 CCS sections from three cell spheroids (five middle sections in one cell spheroid) in each group was performed by using the paired t test.

No	Name	Detection mode	Fold change (Exp/Con, mean ± SD)	*p* value
1	PC(36:1)	P	1.31 ± 0.06	<0.001
2	PC(36:2)	P	1.34 ± 0.15	<0.001
3	PC(34:1)	P	1.68 ± 0.23	<0.001
4	PC(34:4)	P	1.22 ± 0.06	<0.001
5	PC(32:1)	P	1.74 ± 0.24	<0.001
6	SM(d34:1)	P	1.37 ± 0.12	<0.001
7	PI(18:0/22:5)	N	1.63 ± 0.10	<0.001
8	PI(18:0/20:4)	N	1.49 ± 0.04	<0.001
9	PI(18:0/18:1)	N	1.27 ± 0.19	0.0013
10	PE(18:1/20:4)	N	1.26 ± 0.19	<0.001
11	PE(18:1/20:5)	N	1.47 ± 0.25	<0.001
12	PE(18:1p/20:4)	N	1.36 ± 0.19	<0.001
13	PE(18:0/18:1)	N	1.35 ± 0.20	<0.001
14	PE(18:1/18:1)	N	1.35 ± 0.24	<0.001
15	PE(18:1/18:2)	N	1.25 ± 0.25	0.026

16	PE(16:0/20:4)	N	1.40 ± 0.28	<0.001
17	PE(16:0/18:1)	N	1.45 ± 0.11	<0.001

References

1. H. Zhang, X. Shao, H. Zhao, X. Li, J. Wei, C. Yang, Z. Cai, *Environ. Sci. Technol.*, 2019, **53**, 5406–5415.

2. P. Xie, X. Liang, Y. Song, Z. Cai, *Anal. Chem.*, 2020, **92**, 11341–11348.
